# Motivational Biases in Go and Nogo Learning From Immediate Versus Delayed Feedback

**DOI:** 10.1111/ejn.70717

**Published:** 2026-10-26

**Authors:** Julian Vahedi, Sandra Schneider, Livia Swiatek, Jutta Peterburs, Christian Bellebaum

**Affiliations:** ^1^ Faculty of Mathematics and Natural Sciences Heinrich Heine University Düsseldorf Düsseldorf Germany; ^2^ Institute for Systems Medicine and Department of Human Medicine MSH Medical School Hamburg Hamburg Germany

**Keywords:** electroencephalography (EEG), event‐related potentials (ERPs), feedback delay, instrumental learning, motivational biases, Pavlovian bias

## Abstract

Motivational biases in decision‐making describe the phenomenon that individuals are more likely to perform an action to receive a reward and to withhold an action to avoid punishment. These biases likely originate from reward‐related modulations of the striatum, presumably reflecting biased action selection and/or learning, the latter with facilitated learning from rewarded actions and impaired learning from punished inactions. As striatal engagement during learning depends on whether feedback is presented immediately or delayed, the present study examined whether motivational biases are similarly sensitive to feedback timing. Moreover, based on the idea that motivational biases arise through biased learning, we tested whether they manifest as biased feedback processing for action over inaction. Fifty‐six healthy participants performed an orthogonalized go/nogo task learning whether to execute or withhold a response in both reward obtainment and loss‐avoidance contexts—either receiving immediate (*n* = 28) or delayed feedback (*n* = 28). Applying computational modeling of choice behavior and using electroencephalography (EEG), we assessed biased instrumental learning and its neural signature in feedback processing, focusing on the feedback‐related negativity/reward positivity and the P3 in the event‐related potential (ERP). Behavioral results demonstrated robust motivational biases across learning from immediate and delayed feedback. Importantly, motivational biases were best explained in terms of biased action selection rather than biased learning, aligning with our ERP findings that did not provide evidence for action‐biased feedback processing. Taken together, these results highlight motivational biases as a robust hallmark of decision‐making, relying on common mechanisms involved in both learning from immediate and delayed feedback.

## Graphical abstract

Motivational biases have been proposed to originate from the striatum, which is more strongly involved in learning from immediate than delayed feedback. Despite this presumed difference, motivational biases emerged during learning from both immediate and delayed feedback, suggesting they are a hardwired and robust hallmark of decision‐making.

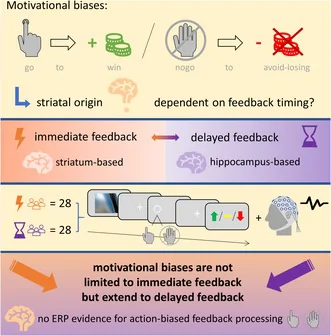

**Abbreviations**
BIC: Bayesian information criterion
BIS/BAS: behavioral inhibition system/behavioral activation system scales
CI: confidence interval
DA: dopamine(rgic)
EEG: electroencephalography
ERP: event‐related potential
FDR: false discovery rate
FRN: feedback‐related negativity
GA: grand average
GAL: go to avoid‐losing
GW: go to win
ICA: independent component analysis
MCC: midcingulate cortex
NGAL: nogo to avoid‐losing
NGW: nogo to win
NLL: negative log likelihood
RewP: reward positivity
RL: reinforcement learning
RPE: reward prediction error
SESOI: smallest effect size of interest
TOST: two one‐sided tests
vEOG/hEOG: vertical/horizontal electro‐oculogram

## 1 Motivational Biases in Go and Nogo Learning From Immediate Versus Delayed Feedback

Decision‐making is a core aspect of human cognition that requires individuals to flexibly evaluate competing response options and their (anticipated) consequences to ensure adaptive and goal‐directed behavior. Everyday life decisions often involve choosing between *action* and *inaction*, that is, executing or withholding a response to achieve a desired outcome. Yet, decision‐making shows a surprising inflexibility, with individuals more likely engaging in action execution to obtain a reward and action suppression to avoid punishment—a phenomenon often referred to as the *Pavlovian bias* (e.g., Cavanagh et al. 2013; Guitart‐Masip et al. 2014; Guitart‐Masip, Huys, et al. 2012; Vahedi et al. 2024).

While the neural underpinnings of this bias are not yet fully understood, converging evidence suggests that it originates from dopaminergic (DA) modulation of the striatum (Guitart‐Masip et al. 2014; also see de Boer et al. 2019; Perosa et al. 2020; Richter et al. 2021; Richter et al. 2014). Seminal work has shown that midbrain DA neurons signal reward prediction errors (RPEs), with phasic bursts in activity for events better (+RPE) and dips below baseline for events worse than expected (−RPE; Bayer and Glimcher 2005; Schultz et al. 1997). In the striatum, +RPE signals are hence theorized to promote action invigoration via the direct (“go”) pathway and −RPE signals to promote action suppression via the indirect (“nogo”) pathway (Collins and Frank 2014; Frank 2005; Frank et al. 2004).

Still, it remains unclear whether the bias reflects (1) a bias on action selection, where the anticipation of reward or punishment—elicited by Pavlovian cues—triggers action invigoration or suppression (Guitart‐Masip, Huys, et al. 2012); (2) biased (instrumental) learning and outcome evaluation, where facilitated learning from rewards following actions may shape the bias that performing an action is more easily associated with reward, while impaired learning from punishments following inactions may underlie the bias that inaction is more easily associated with avoiding punishment (de Boer et al. 2019); or (3) a combination of both (Swart et al. 2017). Recent studies thus referred to such phenomena more generally as *motivational biases* (e.g., Algermissen et al. 2024; Richter et al. 2021; Scholz et al. 2022).

Motivational biases that arise through biased learning may conceivably manifest in feedback processing. A well‐established neural marker of feedback processing is a distinctive fronto‐central negativity in the event‐related potential (ERP) which is more negative for unfavorable compared with favorable feedback (Faßbender et al. 2023; San Martín 2012; Walsh and Anderson 2012). Originally labeled feedback‐related negativity (FRN) and linked to punishment processing (Holroyd and Coles 2002; Miltner et al. 1997), the neural response to unfavorable feedback has recently been interpreted as a baseline, superimposed by a reward positivity (RewP) to favorable feedback (Holroyd et al. 2008; Krigolson 2018; Proudfit 2015). As a consequence, the terms FRN and RewP are often used interchangeably (see Faßbender et al. 2023); hence following Weber and Bellebaum (2024), we use the compound term FRN/RewP.

While the FRN/RewP was found to primarily emerge from the midcingulate cortex (MCC; Hauser et al. 2014; Luu et al. 2003; Oerlemans et al. 2025), it may also reflect striatal processing (Becker et al. 2014; Carlson et al. 2011; Foti et al. 2011). Not surprisingly, the FRN/RewP has been shown to reflect +/−RPEs (Fischer and Ullsperger 2013; Ichikawa et al. 2010; Röhlinger, Albrecht, and Bellebaum 2025; Vahedi et al. 2025; Weber and Bellebaum 2024), whereas other studies suggested the FRN/RewP to only reflect RPE magnitude (Ferdinand et al. 2012; Hauser et al. 2014; Talmi et al. 2013). Notably, motivational biases through biased learning imply differences in the processing of feedback obtained through action versus inaction. Indeed, previous FRN/RewP studies have reported reduced feedback valence coding following inaction (Bikute et al. 2022; Di Bernardi Luft et al. 2024; Yeung et al. 2005). However, these studies did not employ conventional feedback‐based learning task, as (in)actions were implemented as (forced) non‐choices. Hence, although assumed to contribute to motivational biases (Bikute et al. 2022), FRN/RewP evidence from a paradigm explicitly testing such biases and allowing participants to freely choose between action and inaction is still lacking.

Recent work examined whether motivational biases can be modulated by the learning context, but generally found them to be robust and difficult to overcome (e.g., Ereira et al. 2021; Peterburs et al. 2021; Vahedi et al. 2024). While motivational biases may be rooted in the striatum (e.g., de Boer et al. 2019; Guitart‐Masip et al. 2014), the (medial) prefrontal cortex has been linked to mediating or overcoming them (Algermissen et al. 2024; Cavanagh et al. 2013; Kim et al. 2023; Ousdal et al. 2018). Moreover, the hippocampus has been associated with arbitrating approach‐avoidance conflicts (Bach et al. 2014; Bryant and Barker 2020; Le Duc et al. 2025; for a review see Ito and Lee 2016). Thus, modulating motivational biases may require learning contexts that promote more deliberate and reflective processing.

While striatal recruitment during learning is particularly pronounced when feedback is presented immediately, supporting implicit, nondeclarative learning, the medial temporal lobe (including the hippocampus) is increasingly recruited when feedback is delayed by several seconds, supporting more explicit, declarative learning (Foerde and Shohamy 2011; Hinneberg and Hegele 2022). Consistent with this, FRN/RewP feedback valence coding is typically reduced for delayed compared with immediate feedback (Arbel et al. 2017; Höltje and Mecklinger 2018, 2020; Peterburs et al. 2016; Weismüller and Bellebaum 2016). Accordingly, motivational biases may depend on feedback timing, such that learning from delayed feedback may attenuate the impact of striatum‐mediated action biases and shift learning toward more declarative and goal‐directed control. Such a shift may therefore support overriding rigid motivational biases. However, although neural systems for processing immediate and delayed feedback may partly dissociate, they also involve common mechanisms (Weismüller et al. 2018; also see Hinneberg and Hegele 2022), as, for example, (+)RPE encoding in the FRN/RewP was reported for both immediate and delayed feedback processing (Weber and Bellebaum 2024). Whether motivational biases are modulated by feedback timing or rely on common, timing‐invariant learning mechanisms thus remains to be clarified.

The present study aimed to address this gap by examining modulatory influences of feedback timing on motivational biases, while more generally investigating the role of feedback processing underlying them. To this end, electroencephalogram (EEG) was recorded while young healthy adults completed a modified orthogonalized go/nogo task, commonly used to study motivational biases (see Guitart‐Masip et al. 2011), learning from either immediate or delayed feedback. Assuming that learning from delayed feedback recruits more declarative systems possibly promoting more goal‐directed control (Foerde and Shohamy 2011; Hinneberg and Hegele 2022), we hypothesized that motivational biases would be reduced relative to learning from immediate feedback. However, given previous findings highlighting their robustness (e.g., Peterburs et al. 2021) and partially overlapping mechanisms in learning from immediate and delayed feedback (Weber and Bellebaum 2024; Weismüller et al. 2018), motivational biases may also persist across feedback timings. Moreover, we expected motivational biases to reflect biased (instrumental) learning, such that computational modeling of choice behavior should favor a model assuming facilitated learning from rewarded actions and/or impaired learning from punished inactions (see Swart et al. 2017). Biased learning may manifest in the FRN/RewP as stronger feedback valence coding for feedback following action compared with inaction, with increased positivity for rewards following actions (Bikute et al. 2022). This effect may be more strongly pronounced for immediate feedback. Similarly, RPE signaling in the FRN/RewP may be stronger for action relative to inaction, again especially for immediate feedback. Last, in an exploratory analysis, we also assessed later feedback processing, reflected in the P3 that has been linked to coding of motivational significance and outcome magnitude (San Martín 2012; Wu and Zhou 2009; Yeung and Sanfey 2004), for which similarly biased feedback processing was conceivable (see Schevernels et al. 2016).

## 2 Materials and Methods

### 2.1 Participants

Previous ERP studies from our group have shown that sample sizes of approximately 25 participants per experimental group are sufficient to reveal group differences in feedback processing (Bellebaum and Colosio 2014; Weismüller and Bellebaum 2016) and to detect trial‐level RPE encoding (Röhlinger, Albrecht, Ghio, and Bellebaum 2025; Weber and Bellebaum 2024). We therefore targeted a sample size of around 30 participants, which we assumed to provide sufficient statistical power, even after exclusions. Note that comparable sample sizes have also been successfully used to identify group differences in motivational biases at the behavioral level (de Boer et al. 2019; Guitart‐Masip, Chowdhury, et al. 2012).

Accordingly, we recruited 61 healthy adult volunteers through public advertisement and randomly assigned them to perform one of two variants of the orthogonalized go/nogo task: one in which they learned from feedback presented immediately after their choice (*immediate feedback*; *n* = 31) and one from feedback that was delayed by several seconds relative to their choice (*delayed feedback*; *n* = 30). Note that five participants had to be excluded: Two participants (one immediate feedback and one delayed feedback) were excluded as they reported a history of psychiatric disease and/or were on psychotropic medication, two participants (one immediate feedback and one delayed feedback) were excluded after data‐quality control (for details see Section 2.4), and one participant (immediate feedback) was excluded due to technical problems during data recording. Hence, the final sample consisted of 56 participants (28 immediate feedback and 28 delayed feedback; for an overview, see Table 1). Written informed consent was obtained from all participants prior to participation. The study was approved by the Ethical Committee of the Faculty of Mathematics and Natural Sciences at Heinrich Heine University, Düsseldorf (Germany), and was in accordance with the Declaration of Helsinki. A monetary compensation of 20 Euros or course credit was provided for participation.

**TABLE 1:** Sample characteristics after exclusions in the two groups of participants (*N* = 56).

	Immediate feedback (*n* = 28)	Delayed feedback (*n* = 28)^a^
Age (in years)	21.18 (3.94)	21.78 (3.42)
Sex (*n*; female/male)	24/4	22/5
Handedness (*n*; left/right)	23/5	26/1
Verbal IQ	105.46 (8.99)	108.33 (7.09)
BIS^b^	22.48 (3.59)	21.38 (3.14)
BAS^b^	38.78 (3.97)	41.42 (4.34)

*Note:* Data are presented as means with standard deviations (SDs) in parentheses (except for sex and handedness, where data represent the number of cases).

Abbreviations: BAS = behavioral activation system; BIS = behavioral inhibition system; IQ = intelligence quotient.

^a^ Due to a technical error, demographic and questionnaire data of one participant were lost.

^b^ Due to a technical error, BIS/BAS scores of two participants (one immediate feedback and one delayed feedback) were lost.

As motivational biases may partly depend on intelligence (Moutoussis et al. 2018), and to ensure comparable intelligence levels across experimental groups, intelligence was estimated based on a short German multiple‐choice vocabulary test (Mehrfachwahl‐Wortschatz‐Test B, MWT‐B; Lehrl et al. 1995; Merz et al. 1975). Similarly, to assess potential group differences in dispositional reward and punishment sensitivity, which may contribute to motivational biases, the German adaptation of the behavioral inhibition system/behavioral activation system scales (BIS/BAS; Carver and White 1994; Strobel et al. 2001) was employed. There were no significant between‐group differences regarding age (*p* = 0.371), verbal IQ (*p* = 0.110), and BIS scores (*p* = 0.202), whereas BAS scores were slightly increased in the delayed (*M* = 41.42; SD = 4.34) compared with the immediate feedback group (*M* = 38.78; SD = 3.97; *p* = 0.020). Note, however, that neither BIS nor BAS scores covaried with motivational bias strength in a previous study (Vahedi et al. 2024).

### 2.2 Experimental Task and Procedure

Participants completed a modified version of the orthogonalized go/nogo learning task, originally developed by Guitart‐Masip et al. (2011). As illustrated in Figure 1, stimulus sequence and trial timing were structured as follows: Each trial began with one out of four distinct fractal cues (obtained from https://github.com/smathot/materials_for_P0010.5; Mathôt et al. 2015), presented for 1000 ms and followed by a fixation cross for 250–2000 ms. Each cue represented one of four experimental conditions: go to win (GW), go to avoid‐losing (GAL), nogo to win (NGW), and nogo to avoid‐losing (NGAL). After the cue, a white open circle appeared as a target stimulus on either the left or right side of the computer screen for 1000 ms, which served as the response time period. Within this period, participants had to decide whether to respond (*go*) or not respond (*nogo*). If participants chose to respond, they were required to indicate the location of the target stimulus by pressing the left or right Ctrl key on a standard USB keyboard. If participants decided not to respond, they had to simply wait and let the response period pass. The participant's choice was confirmed by presentation of a hand symbol underneath the target stimulus if participants pressed a valid key or a crossed‐out hand symbol if no key was pressed, for 500 ms. Finally, a fixation cross was displayed before participants were shown the outcome of their choice via symbolic feedback. Crucially, experimental groups differed regarding the duration of the fixation cross, thus manipulating feedback timing; that is, the fixation cross was displayed for 500 ms in the immediate feedback variant and 6500 ms in the delayed feedback variant of the task.

**FIGURE 1:**
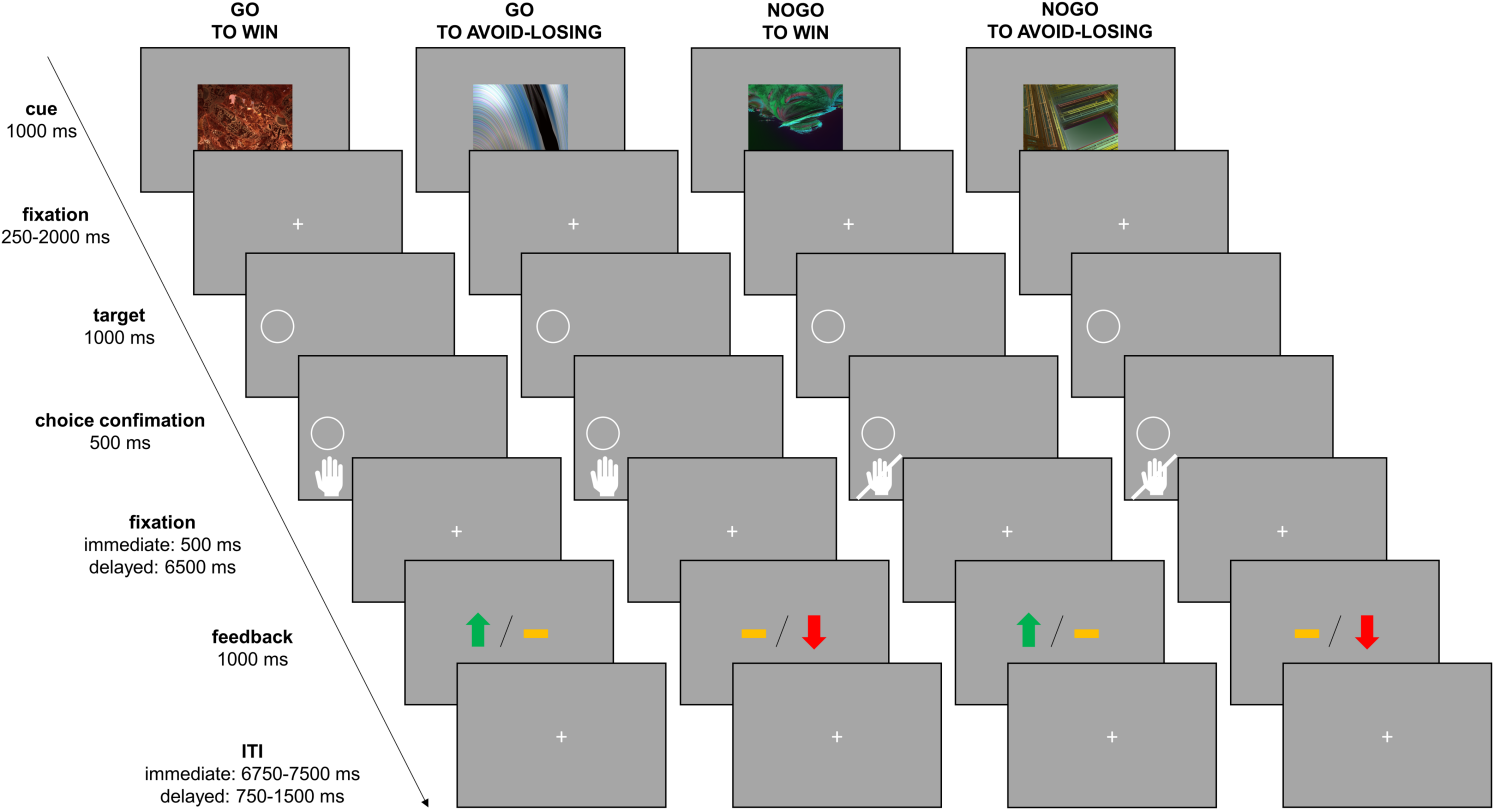
Schematic illustration of the stimulus sequence and timing of an exemplary single trial for each cue condition. In each trial, participants had to either indicate the location of a target circle via pressing a button (go) or omit pressing a button by letting the 1000‐ms response time period pass (nogo). Choices were confirmed by a hand symbol for go choices or a crossed‐out hand symbol for nogo choices. Depending on the feedback timing condition, symbolic feedback was provided either immediately (after 500 ms) or with a slight delay (after 6500 ms) following choice confirmation. In win conditions, the favorable outcome was an upward‐pointing green arrow indicating winning 10 points, while a yellow horizontal bar indicating neither winning nor losing points was the unfavorable outcome. Conversely, in avoid‐losing conditions, the yellow horizontal bar was the favorable outcome indicating avoiding punishment, while a downward‐pointing red arrow indicating losing 10 points was the unfavorable outcome. Feedback validity was 80%. Differences in trial duration for the immediate versus delayed feedback condition were balanced by adjusting the length of the intertrial interval (ITI).

Feedback was presented for 1000 ms as either a green upward‐pointing arrow indicating a gain of 10 points (*win*), a red downward‐pointing arrow indicating a loss of 10 points (*loss*), or a yellow horizontal bar indicating no change in the current score (*draw*). Importantly, for each experimental condition only two outcomes, one favorable and one unfavorable, were possible. In win conditions (GW and NGW), *win* was the favorable outcome whereas *draw* was the unfavorable outcome. By contrast, in avoid‐losing conditions (GAL and NGAL), *draw* was the favorable outcome whereas *loss* was the unfavorable outcome. Therefore, *draw* feedback could be received in each of the four cue conditions, while *win* and *loss* feedback, respectively, could only be received in two of the four cue conditions while never co‐occurring in the same cue condition (i.e., *win* only in GW/NGW and *loss* only in GAL/NGAL). Choices were classified as correct when they matched the *required response* for a given cue (i.e., *go* on GW/GAL and *nogo* on NGW/NGAL) and were hence associated with the best possible outcome. Crucially, feedback was probabilistic with a validity of 80%, such that correct responses were followed by favorable outcomes on 80% of trials, while incorrect responses resulted in favorable outcomes on 20% of trials. Participants were not explicitly informed about the probabilistic nature of the task. Notably, performance feedback was only presented when participants made a valid choice. Choices were considered invalid if participants pressed the key opposite the target location or responded outside the response period (> 1000 ms) during choice confirmation. In such cases, participants were reminded to carefully follow the task instructions. Participants were instructed to collect as many points as possible by maximizing favorable and minimizing unfavorable outcomes.

To ensure comparable trial length in both feedback timing variants and prevent inequal task durations to confound the results, we adjusted the duration of the intertrial interval. Accordingly, a fixation cross with a jittered duration of 6750–7500 ms was presented in the immediate feedback group, whereas a jittered duration of 750–1500 ms was used in the delayed feedback group. Thus, task completion took about 35 min for both groups. The experiment consisted of four blocks of 40 trials, that is, a total of 160 trials. Presentation software (Version 20.1, Neurobehavioral Systems Inc., Albany, CA, USA) was used to control the experimental task and recording of behavioral responses.

### 2.3 EEG Acquisition and Data Processing

#### 2.3.1 EEG Acquisition

EEG signals were acquired at a 1000‐Hz sampling rate using a Brain Amp DC amplifier (Brain Products GmbH, Gilching, Germany) and BrainVision Recorder software (Version 1.20.0506, Brain Products GmbH, Gilching, Germany). Twenty‐nine active silver/silver‐chloride electrodes (Fz, F3, F4, F7, F8, FCz [= on‐line reference], FC1, FC2, FC5, FC6, Cz, C3, C4, T7, T8, CP1, CP2, CP5, CP6, Pz, P3, P4, P7, P8, PO9, PO10, Oz, O1, and O2) were attached to an actiCAP elastic cap (Brain Products GmbH, Gilching, Germany) in accordance with the extended 10–20 system (Chatrian et al. 1985, 1988). Moreover, two electrodes were attached, one to each mastoid, for off‐line re‐referencing, and two additional electrodes were placed above the left eye and on the left outer canthus to record the vertical/horizontal electro‐oculogram (vEOG/hEOG). The ground electrode was positioned at AFz. Impedances were kept below 20 kΩ.

#### 2.3.2 Preprocessing

EEG preprocessing was performed using BrainVision Analyzer software (v2.2.0; Brain Products GmbH, Gilching, Germany). In a first step, raw EEG recordings of each participant were visually inspected to identify defective or overly noisy channels. If present, any such channels were interpolated and the original signal removed (mean number of interpolated channels: 0.13, SD = 0.38, range = 0–2). Next, EEG data were re‐referenced to the linked mastoids, thus allowing signal recovery of electrode site FCz. The re‐referenced EEG data were then filtered using a 0.1‐ to 30‐Hz bandpass and 50‐Hz notch filter. To mitigate ocular artifacts, we used BrainVision Analyzer's built‐in ocular correction independent component analysis (ICA) algorithm in semiautomatic mode (vEOG/hEOG against common average; Plank 2013).

The continuous EEG data were subsequently segmented into epochs of 800‐ms length, spanning from 200 ms before to 600 ms after feedback onset. Because biased feedback processing should specifically occur for win and loss feedback, only segments corresponding to these non‐neutral outcomes were considered in this study. Baseline correction was then applied by subtracting the mean voltage of the pre‐feedback interval from each time point of the epoch. Ultimately, automatic artifact rejection excluded segments according to the following criteria: extreme amplitude fluctuations (exceeding ±100 μV), rapid voltage changes (> 200 μV within 200 ms), abrupt voltage steps (> 50 μV between consecutive data points), or low signal activity (< 0.5 μV within 100 ms). On average, applying these criteria led to the exclusion of 1.33% of segments (SD = 2.62). The remaining clean epochs were exported to MATLAB (Version 9.8, R2020a; The MathWorks Inc., Natick, Massachusetts, USA) for further data processing.

#### 2.3.3 ERP Component Scoring Procedure

The FRN/RewP was measured from electrodes within a fronto‐central cluster (Fz, FC1, FC2, and FCz). For exploratory P3 analyses, the P3 was measured from electrodes within a centro‐parietal cluster (Cz, CP1, CP2, and Pz; see the Supporting Information for results on the P3 measured within a fronto‐central cluster). Both the FRN/RewP and the P3 were scored separately for the immediate versus delayed feedback group, consistent with Weber and Bellebaum (2024). The scoring procedure was performed in accordance with Vahedi et al. (2025): For each electrode cluster (and feedback timing condition), we first created a composite waveform, calculated as the unweighted mean of the win‐ and loss‐related grand average (GA) signal, pooled across electrodes within its cluster.

$$\text{COMP}=\frac{\text{pooled}\;{\mathrm{GA}}_{\mathrm{win}}+\text{pooled}\;{\mathrm{GA}}_{\text{loss}}}{2}$$

Next, this composite waveform was used to identify the peak latency of each ERP component within a literature‐backed a priori search window. Specifically, the FRN/RewP peak was searched as the local minimum in the time window 200‐ to 350‐ms postfeedback (Faßbender et al. 2023). Conversely, the P3 peak was searched as the local maximum in the time window 250‐ to 500‐ms postfeedback (Polich 2007). Each ERP component was eventually scored as the mean amplitude ±30 ms around the identified peak latency, for each individual electrode within its cluster, on each single trial. Details about empirical extraction time windows used for each ERP component are provided in Table 2.

**TABLE 2:** Scoring procedure.

ERP component	Cluster	Search window	Extraction time window
FRN/RewP	Fronto‐central (Fz, FC1, FC2, and FCz)	200–350 ms^a^	Immediate feedback: 217–277 ms; delayed feedback: 242–302 ms
P3	Centro‐parietal (Cz, CP1, CP2, and Pz)	250–500 ms^b^	Immediate feedback: 325–385 ms; delayed feedback: 335–395 ms

Abbreviations: ERP = event‐related potential; FRN/RewP = feedback‐related negativity/reward positivity.

^a^ FRN/RewP search window was based on Faßbender et al. (2023).

^b^ P3 search window was based on Polich (2007).

### 2.4 Data‐Quality Control

Following Vahedi et al. (2024) and to prevent including participants with non‐compliance and/or low motivation, we examined choice data based on three data‐quality criteria for exclusion: First, we excluded participants with choice accuracy below‐chance level (according to a one‐tailed binomial test against chance, *α* = 0.05) in GW, that is, the easiest learning condition (also see Albrecht et al. 2016; Weber et al. 2022). Second, we excluded participants with > 25% invalid key presses (i.e., key press on wrong side or late responding outside the response period) as in those trials no feedback was presented and participants could hence not learn the reward contingencies (Scholz et al. 2022). Last, we excluded participants who were overly biased by choosing the same response (go or nogo) in > 85% of trials (similar to Weber et al. 2022). Based on these criteria, two participants (one immediate feedback and one delayed feedback) with below‐chance GW learning were excluded from further analyses. Furthermore, we removed invalid trials, with a total 3.40% of trials being removed.

### 2.5 Computational Modeling of Behavioral Data

#### 2.5.1 Model Building

We fitted a series of Rescorla–Wagner‐style reinforcement learning (RL) models (Sutton and Barto 2018) aiming to dissociate latent variables and test for the presence of an instrumental learning bias. In all models, state‐action weights $W_{t}\left(a_{t},s_{t}\right)$ were estimated for each choice a (*go* and *nogo*) and cue s (*GW*, *GAL*, *NGW*, and *NGAL*) on every trial t and converted into choice probabilities p using a softmax function. Choice probabilities $p\left(a_{t}|s_{t}\right)$ were calculated using an inverse temperature parameter $\beta \in \left(0,+\infty \right)$, capturing decision noise, where higher values of β reflected more deterministic and lower values of β led to more random decision‐making, that is, exploitation and exploration, respectively:

$$p\left(a_{t}|s_{t}\right)=\frac{\exp\left(\beta *W_{t}\left(a_{t},s_{t}\right)\right)}{\sum_{a^{\prime }}\exp\left(\beta *W_{t}\left(a^{\prime },s_{t}\right)\right)}$$

In the most parsimonious model M0, $W_{t}\left(a_{t},s_{t}\right)$ equaled learned instrumental state‐action values $Q_{t}\left(a_{t},s_{t}\right)$, which reflected the anticipated reward associated with a given choice for a given cue. Q values were updated based on feedback $r\in \left\{-1,0,1\right\}$, indicating loss, draw, and win, respectively, and a learning rate parameter $\varepsilon \in \left[0,1\right]$.

$$Q_{t}\left(a_{t},s_{t}\right)=Q_{t-1}\left(a_{t},s_{t}\right)+\varepsilon \;\left(r_{t}-Q_{t-1}\left(a_{t},s_{t}\right)\right)$$

In accordance with previous studies (e.g., Guitart‐Masip, Chowdhury, et al. 2012; Raab and Hartley 2020; Swart et al. 2017), M0 was incrementally extended by adding additional model parameters. In M1, we first added a go bias parameter $b\in \left(-\infty ,+\infty \right)$, with positive values of b indicating a bias toward go and negative values a bias toward nogo responding. Hence, in M1, $W_{t}\left(a_{t},s_{t}\right)$ was calculated as

$$W_{t}\left(a_{t},s_{t}\right)=\begin{array}{cc} Q_{t}\left(a_{t},s_{t}\right)+b & \text{if}\;\mathrm{go}\\ \;Q_{t}\left(a_{t},s_{t}\right)\; & \text{else}\; \end{array}$$

In M2, we additionally implemented a Pavlovian bias parameter $\pi \in \left(-\infty ,+\infty \right)$. This bias was added as a bonus term by scaling the Pavlovian state value $V_{t}\left(s_{t}\right)$, reflecting reward expectation associated with the cue, but independent of any choice:

$$W_{t}\left(a_{t},s_{t}\right)=\begin{array}{cc} Q_{t}\left(a_{t},s_{t}\right)+b+\pi V_{t}\left(s_{t}\right) & \text{if}\;\mathrm{go}\\ \;Q_{t}\left(a_{t},s_{t}\right)\; & \text{else}\; \end{array}$$

In analogy to $Q_{t}\left(a_{t},s_{t}\right)$, V values were updated as follows:

$$V_{t}\left(s_{t}\right)=V_{t-1}\left(s_{t}\right)+\varepsilon \left(r_{t}-V_{t-1}\left(s_{t}\right)\right)$$

Following recent studies suggesting a role of biased instrumental learning (e.g., Algermissen et al. 2024; de Boer et al. 2019; Swart et al. 2017), M3–M5 contained different parametrizations of an instrumental bias parameter $k\in \left(-\infty ,+\infty \right)$. At the conceptual level, in all of these models, k modulated the learning rate ε by acting as a bonus weight for rewarded go choices and/or as a penalty weight for punished nogo choices, that is,

$$\varepsilon =\left\{\begin{array}{cc} \varepsilon +k & \text{if}\;a=\mathrm{go}\;\text{and}\;r=\mathrm{win}\;\\ \varepsilon -k & \text{if}\;a=\text{nogo and}\;r=\text{loss}\\ \varepsilon & \text{else}\; \end{array}\right.$$

Because adding or subtracting k could push ε outside its mathematical bounds (i.e., [0, 1]), ε was therefore optimized in an unconstrained space, that is, $\varepsilon \in \left(-\infty ,+\infty \right)$, but mapped onto [0, 1] via inverse logit transformation, that is, $\varepsilon_{0}$ (Algermissen et al. 2024; Swart et al. 2018; Swart et al. 2017). To ensure that k had a symmetric effect after this transformation, we first checked whether $\varepsilon_{0}$ was below or above 0.5. We then computed one part of the bias (i.e., $\varepsilon_{\text{punishedNogo}}$ if $\varepsilon_{0}$ was close to 0 or $\varepsilon_{\text{rewardedGo}}$ if $\varepsilon_{0}$ was close to 1) and applied the same‐sized change in the opposite direction for the other part of the bias to keep it balanced, that is,

$$\varepsilon =\left\{\begin{array}{cc} \varepsilon_{0}=\mathrm{inv}.\text{logit}\left(\varepsilon \right) & \;\\ \varepsilon_{\text{punishedNogo}}=\mathrm{inv}.\text{logit}\left(\varepsilon -k\right) & \text{if}\;\varepsilon_{0}<0.5\\ \varepsilon_{\text{rewardedGo}}=\varepsilon_{0}+\left(\varepsilon_{0}-\varepsilon_{\text{punishedNogo}}\right) & \;\text{if}\;\varepsilon_{0}<0.5\; \end{array}\right.$$

$$\varepsilon =\left\{\begin{array}{cc} \varepsilon_{0}=\mathrm{inv}.\text{logit}\left(\varepsilon \right) & \;\\ \varepsilon_{\text{rewardedGo}}=\mathrm{inv}.\text{logit}\left(\varepsilon +k\right) & \text{if}\;\varepsilon_{0}>0.5\\ \varepsilon_{\text{punishedNogo}}=\varepsilon_{0}-\left(\varepsilon_{\text{rewardedGo}}-\varepsilon_{0}\right) & \;\text{if}\;\varepsilon_{0}>0.5\; \end{array}\right.$$

Accordingly, in M3, the learning rate ε was modulated by both rewarded go and punished nogo choices by adding or subtracting the instrumental learning bias k, respectively. Moreover, and similar to de Boer et al. (2019), we also tested models that *only* added a bonus weight for rewarded go choices ($k_{\text{rewardedGo}}$ M4) or subtracted a penalty weight for punished nogo choices ($k_{\text{punishedNogo}}$; M5). Finally, in models M6–M8, we included both the Pavlovian bias parameter π and one of the three parametrizations of the instrumental bias parameter (i.e., k, $k_{\text{rewardedGo}}$, and $k_{\text{punishedNogo}}$) in the same model. For all models, Q and V values were initialized at the value of 0 (i.e., neither expecting reward nor loss; but see the Supporting Information for modeling with alternative initialization values).

#### 2.5.2 Model Fitting and Comparison

Models were fitted using a maximum a posteriori approach via the *fmincon* function (from the Optimization Toolbox) in MATLAB (see, e.g., Raab and Hartley 2020). Accordingly, we used minimally informative priors to regularize parameter ranges and to improve parameter identifiability (see Daw et al. 2011; Raab and Hartley 2020). A prior of *beta*(1.1, 1.1) was used for the ε parameter in models M0–M2 and a prior of *normal*(0, 1) for ε in models M3–M8. In addition to that, a prior of *normal*(0, 1) was also used for parameters b, π, k (including $k_{\text{rewardedGo}}$ and $k_{\text{punishedNogo}}$), whereas a prior of *gamma*(1.2, 5) was used for β. To reduce the risk of converging to local optima, optimization for each model and participant was initiated from 250 randomly chosen starting points. Models were compared to determine the winning model that best accounted for participants' observed behavior, based on the negative log likelihood (NLL), computed from choice probabilities $p\left(a_{t}|s_{t}\right)$, that is,

$$\mathrm{NLL}=-\sum_{t}\log\left(p\left(a_{t}|s_{t}\right)\right)$$

with the Bayesian information criterion (BIC) calculated as

$$\mathrm{BIC}=2\mathrm{NLL}+n_{\text{params}}\;\log\left(N\right)$$

where $n_{\text{params}}$ refer to the number of free model parameters and N to the number of observations. The winning model was considered the model with the lowest BIC value, that is, indicating the best model fit.

#### 2.5.3 Model Validation

Validation of the winning model included examining parameter identifiability and parameter recovery (Palminteri et al. 2017; Wilson and Collins 2019). Parameter identifiability was assessed via intercorrelations, where Pearson's correlation coefficients |*r*| ≤ 0.50 were considered indicative of good identifiability. To evaluate parameter recovery, the winning model was re‐fitted to 25 synthetic datasets per participant, generated from the parameter estimates obtained from the actual experimental data. Recovery success was quantified by computing Pearson's correlation coefficients between the true (empirical) and recovered (synthetic) parameter values, with *r* ≥ 0.70 considered indicative of good recovery. To reduce variability, recovered parameters were averaged across simulations for each participant.

### 2.6 Statistical Analysis

Behavioral and ERP data were analyzed at the single‐trial level via (generalized) linear mixed‐effects models ([G]LMMs), using the *lme4* package (Bates et al. 2015) in R (version 4.1.0; R Core Team 2021). In all models, (categorical) fixed effect predictors were coded using deviation contrasts (−0.5 vs. 0.5). To account for by‐participant random variation, we implemented the maximal random‐effects structure permitted by the data (Barr et al. 2013), determined via the *buildmer* function from the eponymous R package (Voeten 2020). ERP models additionally incorporated a random intercept by electrode. Full model formulae are provided in Wilkinson notation together with detailed modeling results in the Supporting Information. The *bobyqa* algorithm was used for parameter optimization with a limit of 1e6 function evaluations. For GLMMs, *p*‐values were derived from Wald *z*‐tests (but reported as equivalent *χ* ^2^‐tests), and for LMMs based on Satterthwaite's approximation for degrees of freedom, as implemented in the *lmerTest* package (Kuznetsova et al. 2017). Effects with *p < 0*.050 were considered statistically significant. The *emmeans* package (Lenth 2022) was used to probe significant interactions, with *p*‐values adjusted for multiple comparisons using the false discovery rate (FDR; Benjamini and Hochberg 1995).

In case of non‐significant differences between conditions relevant to our hypotheses, we performed equivalence testing following the two one‐sided tests (TOST) rule (Lakens 2017; Lakens et al. 2018), implemented in the *parameters* package (Lüdecke et al. 2020). This approach evaluates whether an observed effect is statistically distinguishable from effects larger than a predefined smallest effect size of interest (SESOI) by testing whether its 90% confidence interval (CI) lies entirely within a region of practical equivalence (i.e., ±SESOI). We chose a SESOI of ±0.18 log odds for choice behavior (Van der Biest et al. 2025; also see Maxwell et al. 2015) and ±0.50 μV for EEG measures, corresponding to a small but potentially meaningful effect in the ERP (see Jensen and MacDonald 2023). Crucially, equivalence testing was preferred over selective Bayes factor analyses for non‐significant effects, which can be biased toward the null hypothesis (Schreiner and Kunde 2024) and is inconsistent with recent advice to maintain a single inferential framework (Dienes 2024).

#### 2.6.1 Behavioral Data

Motivational biases in decision‐making were examined using GLMM analysis of choice accuracy. Fixed effects included the within‐subject predictors cue valence (*win [= reference]* and *avoid‐losing*) and required response (*go [= reference]* and *nogo*) as well as the between‐subject predictor feedback timing (*immediate [= reference]* and *delayed*). Choice accuracy (0/1 = correct/incorrect) was set as a binary logistic dependent variable. The presence of motivational biases was operationalized as the effect of a cue valence × required response interaction.

In addition to RL modeling, biased learning was also exploratively investigated using a model‐free approach. Therefore, a second GLMM assessed choice repetition behavior when encountering the same cue again (0/1 = switch/repeat), similar to win‐stay/lose‐shift behavior (Algermissen et al. 2024; also see Scholz et al. 2024). Because biased learning should primarily affect learning from *true* win and loss outcomes (Algermissen et al. 2024), we decided to omit trials with neutral outcomes for this analysis. Fixed effects included the within‐subject predictors previous outcome valence (*win [= reference]* and *loss*) and response type (*go [= reference]* and *nogo*) as well as the between‐subject predictor feedback timing (*immediate [= reference]* and *delayed*). Note that in contrast to the first GLMM, response type now reflects the *actual* choice made by the participant (on the previous trial of the same cue). Accordingly, an instrumental learning bias should manifest as the previous outcome valence × previous response type interaction, with a stronger effect of outcome valence for go compared with nogo choices.

#### 2.6.2 EEG Data

Feedback processing was assessed using LMMs, with FRN/RewP and the feedback‐P3 amplitude as the dependent variable, respectively. Fixed effects included the categorical between‐subject predictor feedback timing (*immediate [= reference]* and *delayed*), as well as the within‐subject predictors outcome valence (*win [= reference]* and *loss*) and response type (*go [= reference]* and *nogo*). With these predictors, biased feedback processing, as predicted by an instrumental learning bias, should manifest as the response type × outcome valence interaction. Importantly, to fully disentangle effects of expectancy from those of outcome valence, we sought to investigate RPE coding. Trial‐level RPEs were decomposed into its two constituents, that is, the expected state‐action value (Q) and the received outcome (Burnside et al. 2019; Fischer and Ullsperger 2013; also see Caplin and Dean 2008). Single‐trial Q values were obtained via latent variable inference from the winning RL model (Wilson and Collins 2019). Therefore, we included the absolute value of Q (i.e., ∣Q∣) as an additional continuous predictor. Feedback following low ∣Q∣ was weakly expected (i.e., unexpected), whereas feedback following high ∣Q∣ was strongly expected, such that a main effect of ∣Q∣ served as a proxy for the encoding of RPE magnitude, which has been linked to both the FRN/RewP (Ferdinand et al. 2012; Hauser et al. 2014; Talmi et al. 2013; Walentowska et al. 2019) and feedback‐P3 (Kirsch et al. 2022; Philiastides et al. 2010). Encoding of signed RPEs was indicated by interaction of its constituents, that is, a significant outcome valence × ∣Q∣ interaction. The higher order response type × outcome valence × ∣Q∣ interaction would hence capture differences between RPE encoding for action versus inaction, although note that in case the winning RL model included an instrumental learning bias, RPEs itself would already be action biased. Trial‐level ∣Q∣ values were centered around 0.5 prior to model fitting.

We ensured to include a minimum of 10 trials (see Bellebaum and Daum 2008; Ichikawa et al. 2010) for each outcome valence × response type combination (4.48% of trials excluded, mean number of trials = 21.65, SD = 8.36, range = 10–40). Importantly, note that using LMMs improves parameter estimation even if including cases with limited data through partial pooling, which effectively reduces bias from low‐trial observations (see, e.g., Gomes 2022).

## 3 Results

### 3.1 Computational Modeling

Model comparison revealed that M2 was the best fitting model as indicated by the lowest (mean) BIC (see Figure 2a). M2 included a learning rate ε, a go bias b, a Pavlovian bias parameter π, and an inverse temperature parameter β. Although mean BIC scores across models were relatively close (see Figure 2a), examining the sum of BICs across participants as an index of accumulated model evidence (Li et al. 2008) confirmed M2 as the best fitting model. Specifically, M2 was favored over the second‐best model M3 that included an instrumental rather than Pavlovian bias parameter, with ΔBIC_mean_ = 2.55 and ΔBIC_sum_ = 142.75. Accordingly, choice behavior was overall better explained by a Pavlovian response bias rather than an instrumental learning bias. Still, note that there was some heterogeneity in individual model fits (see Table S1), suggesting that (latent) computational mechanisms may have slightly differed between participants.

**FIGURE 2:**
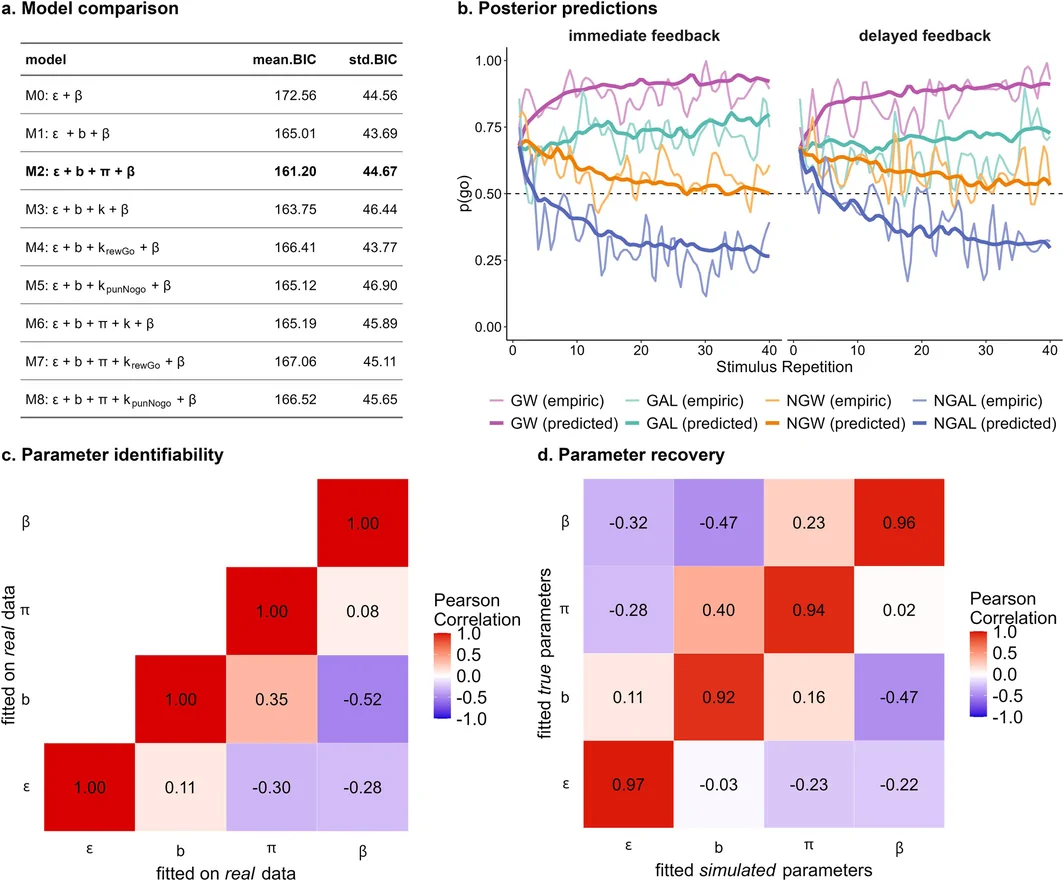
Computational modeling results. (a) Model comparisons with the winning model highlighted in bold. (b) Posterior predictions for the winning model M2 with smoothed empirical data and (using locally estimated scatterplot smoothing) in lighter color intensity. (c,d) Results from validating the winning model M2, that is, parameter identifiability and parameter recovery. Model parameters: *ε* = learning rate; *b* = go bias parameter; *π* = Pavlovian bias parameter; *k* = instrumental learning bias parameter; *β* = inverse temperature parameter. BIC = Bayesian information criterion; GAL = go to avoid‐losing; GW = go to win; NGAL = nogo to avoid‐losing; NGW = nogo to win.

Model validation of M2 revealed that all parameters were generally identifiable (all |*r*| ≤ 0.52; see Figure 2c), and parameter recovery analysis further revealed excellent recoverability (all *r* ≥ 0.92), with no spuriously high intercorrelations (all |*r*| ≤ 0.47; see Figure 2d). Comparing parameter estimates between feedback timings did not show any significant differences (all *p*s ≥ 0.499).

### 3.2 Behavioral Data

A detailed summary of inferential statistics of behavioral GLMM analyses is provided in Tables S4 and S5.

#### 3.2.1 Choice Accuracy

GLMM analysis of choice accuracy yielded significant main effects of cue valence, *b* = −0.37 (SE = 0.15), *χ* ^2^(1) = 5.76, *p* = 0.016, and required response, *b* = −1.80 (SE = 0.17), *χ* ^2^(1) = 109.16, *p* < 0.001, suggesting overall better learning performance in the win versus the avoid‐losing context and when the required response was go versus nogo. Crucially, motivational biases were evident as reflected in a significant cue valence × required response interaction effect, *b* = 2.96 (SE = 0.39), *χ* ^2^(1) = 57.25, *p* < 0.001. Resolving this effect confirmed better learning in GW compared with GAL, *b* = −1.85 (SE = 0.23), *z* = −7.87, *p*_adj_ < 0.001, and NGAL compared with NGW, *b* = 1.11 (SE = 0.26), *z* = 4.24, *p*_adj_ < 0.001. Importantly, there were no significant main or interaction effects involving the predictor feedback timing (*p* ≥ 0.311), providing no evidence that feedback timing modulated motivational biases (see Figure 3a) or choice behavior more generally. To formally assess whether motivational bias strength was practically equivalent across feedback timings, we performed equivalence testing on the originally non‐significant feedback timing × cue valence × required response interaction. The effect (90% CI = [−1.55, 0.95]) overlapped with but did not lie entirely within the region of practical equivalence ([−0.18, 0.18]), *p* = 0.825; thus, we could not provide evidence for the practical absence of a feedback timing effect on motivational biases. Moreover, as this was central to our hypothesis, we conducted subordinate exploratory GLMM analyses separately for feedback timing, to examine whether motivational biases emerged independently across feedback timings (see Weber and Bellebaum 2024 for a similar approach). This was supported by significant cue valence × required response interactions for both immediate, *b* = 3.09 (SE = 0.57), *χ* ^2^(1) = 29.74, *p* < 0.001, and delayed feedback, *b* = 2.88 (SE = 0.56), *χ* ^2^(1) = 26.08, *p* < 0.001.

**FIGURE 3:**
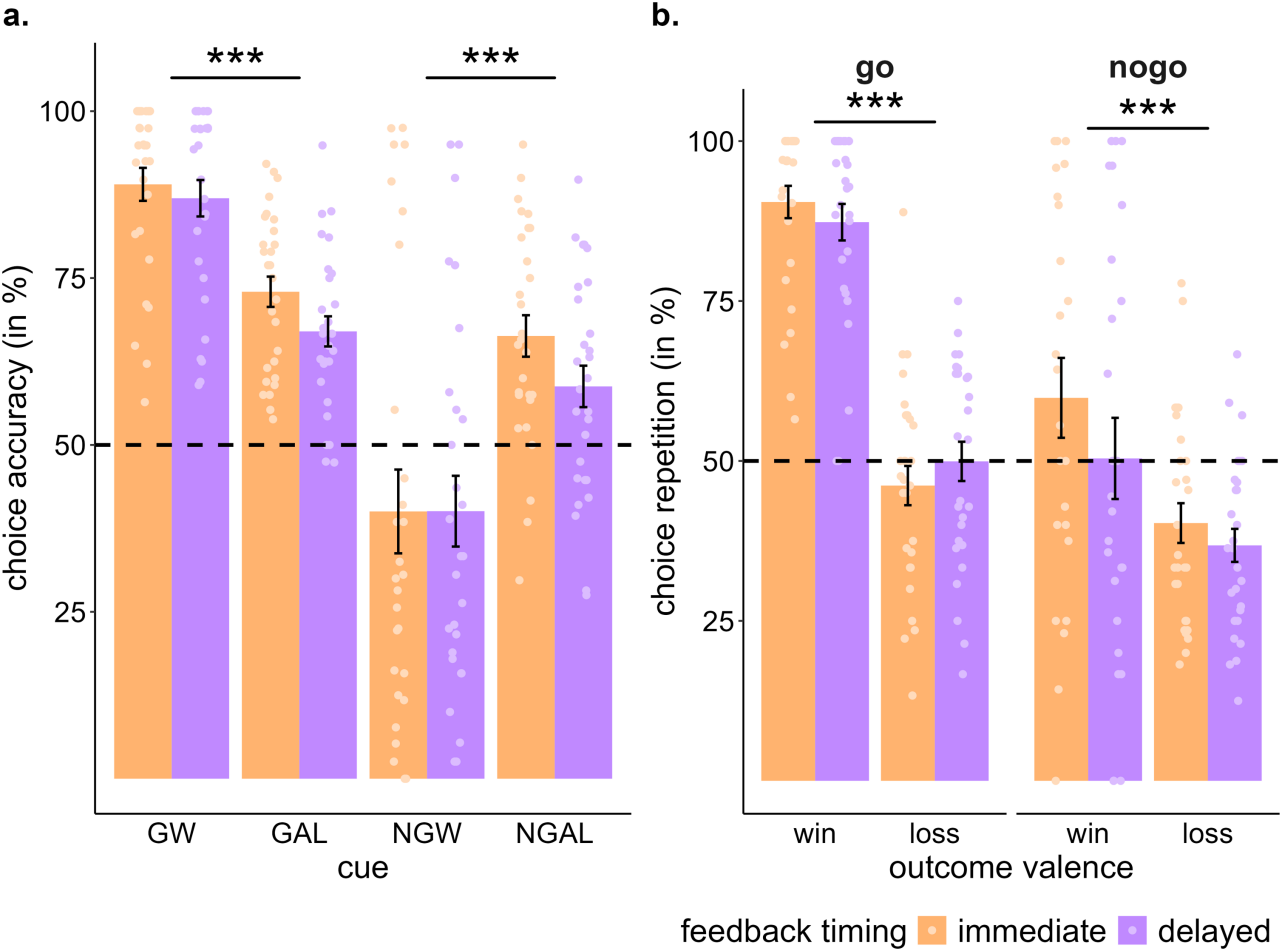
(a) Task performance for all combinations of required response and cue valence (GW = go to win; GAL = go to avoid‐losing; NGW = nogo to win; NGAL = nogo to avoid‐losing), separately for each feedback timing group. (b) Choice repetition behavior for all combinations of previous outcome valence and previous response type, separately for each feedback timing group. Both plots are based on descriptive data. Lighter‐shaded dots indicate mean choice accuracies at the subject level. Error bars represent the standard error of the mean. Asterisks indicate significant differences yielded from simple effects analyses. The dotted black line represents chance level performance and repetition behavior (= 50%), respectively. ·< 0.100, *p < 0.050, **p < 0.01, ***p < 0.001.

#### 3.2.2 Choice Repetition

The second, explorative GLMM for choice repetition behavior revealed significant main effects of previous outcome valence, *b* = −2.17 (SE = 0.23), *χ* ^2^(1) = 92.21, *p* < 0.001, and previous response type, *b* = −1.12 (SE = 0.12), *χ* ^2^(1) = 88.84, *p* < 0.001. Hence, participants were more likely to repeat their previous choice following wins than losses and following go than nogo choices. Moreover, the previous outcome valence × previous response type interaction proved to be significant, suggesting biased learning, with a larger effect of outcome valence on choice repetition behavior following go compared with nogo choices, *b* = 1.42 (SE = 0.19), *χ* ^2^(1) = 55.22, *p* < 0.001 (see Figure 3b). Importantly, further resolving this effect showed that participants showed increased repetition of go versus nogo choices following wins, *b* = 1.83 (SE = 0.18), *z* = 10.45, *p*_adj_ < 0.001, in line with the assumed facilitation of learning from rewarded actions. Conversely, choice repetition was decreased for nogo versus go choices following losses, *b* = 0.41 (SE = 0.13), *z* = 3.27, *p*_adj_ = 0.001, contrary to the presumed impairment of learning from punished inactions. Again, there were no significant effects involving the predictor feedback timing (*p* ≥ 0.145), providing no evidence that feedback timing modulated the instrumental learning bias or choice repetition behavior more generally. This was further confirmed by subordinate exploratory GLMM analyses separately for feedback timing, showing that the previous outcome valence × previous response type interaction emerged independently of learning from both immediate, *b* = 1.59 (SE = 0.29), *χ* ^2^(1) = 30.53, *p* < 0.001, and delayed feedback, *b* = 1.27 (SE = 0.25), *χ* ^2^(1) = 25.21, *p* < 0.001. Lastly, equivalence testing on the non‐significant previous feedback timing × outcome valence × previous response type interaction revealed that the effect (90% CI = [−0.91, 0.32]) overlapped with but did not lie entirely within the region of practical equivalence ([−0.18, 0.18]), *p* = 0.720; consequently, we could not provide evidence for the practical absence of this effect.

### 3.3 EEG

Feedback‐locked GA ERP waveforms are presented in Figure 4, with scalp topographies underlying the ERP results reported below shown in Figure S2. A detailed overview of inferential statistics for all LMM analyses is provided in Tables S6–S9.

**FIGURE 4:**
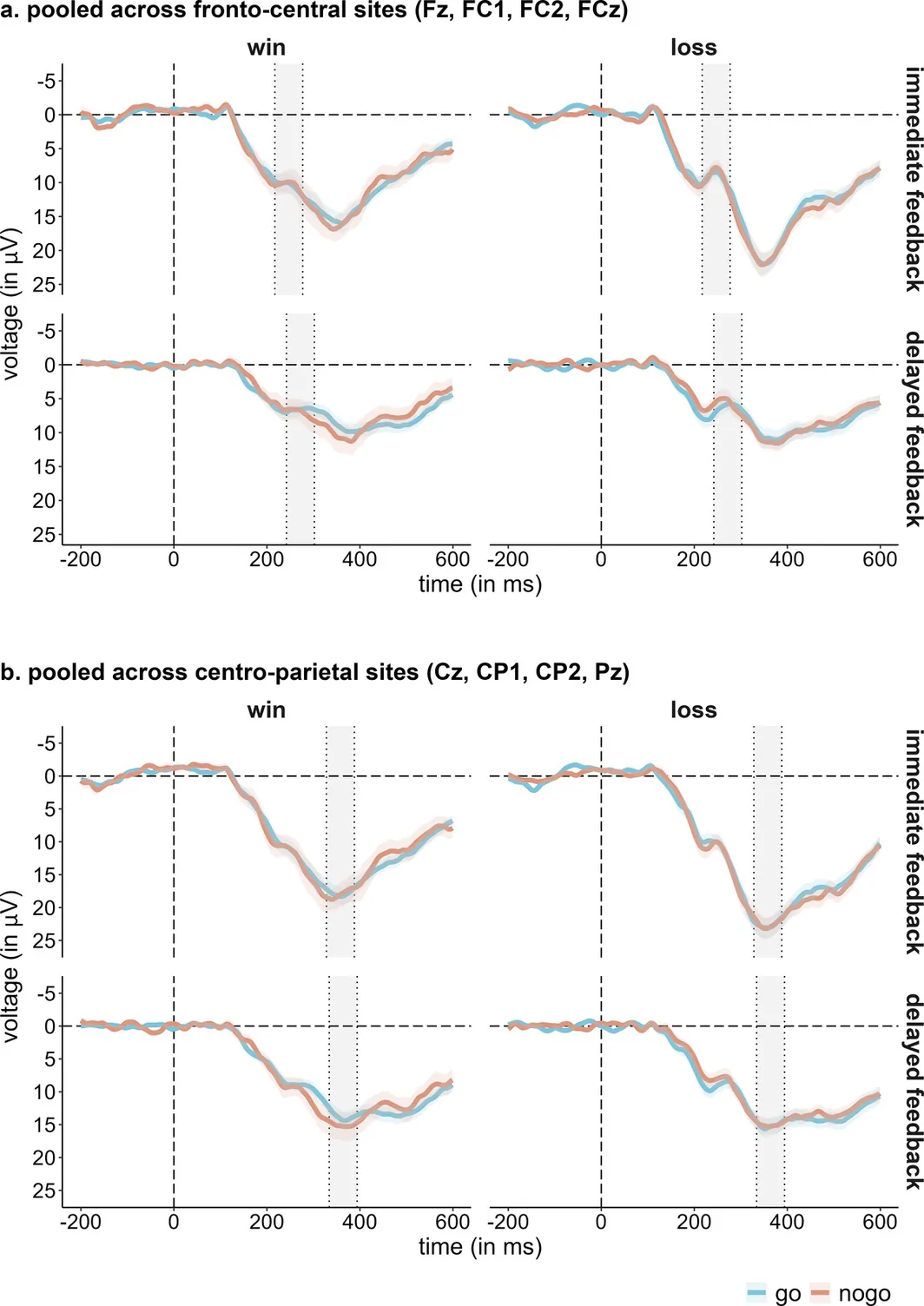
Feedback‐locked grand average ERP waveforms. Data are presented as mean ± standard error. Gray‐shaded areas indicate the measurement windows used to score the feedback‐related negativity/reward positivity (FRN/RewP) (shown in a) and the feedback‐P3 (shown in b). ERP = event‐related potential.

#### 3.3.1 FRN/RewP

FRN/RewP results revealed a significant main effect of feedback timing, *b* = −5.00 (SE = 1.97), *F*(1, 38.95) = 6.48, *p* = 0.015, indicating that amplitudes were overall more negative for delayed versus immediate feedback. The effect of feedback timing was further qualified by a significant feedback timing × ∣Q∣ interaction effect, *b* = −7.28 (SE = 2.98), *F*(1, 28.27) = 5.97, *p* = 0.021, indicating that the FRN/RewP was more negative when feedback was more unexpected, for immediate feedback, *b* = 6.46 (SE = 2.13), *t*(28.90) = 3.04, *p*_adj_ = 0.010, but not for delayed feedback (*p*_adj_ = 0.696). Moreover, also, the outcome valence × ∣Q∣ interaction was significant, *b* = 7.46 (SE = 1.60), *F*(1, 31.26) = 21.79, *p* < 0.001, suggesting that, overall, the FRN/RewP reflected −RPEs, *b* = 6.54 (SE = 1.95), *t*(27.67) = 3.35, *p*_adj_ = 0.005, but no +RPEs (*p*_adj_ = 0.513). Further resolving this interaction using a spotlight approach indicated that the FRN/RewP was more negative for losses versus wins only when feedback was unexpected (∣Q∣ = −0.25), *b* = −1.39 (SE = 0.61), *t*(39.27) = −2.27, *p*_adj_ = 0.038, whereas the opposite, that is, a more positive FRN/RewP for losses versus wins was observed when feedback was expected (∣Q∣ = 0.25), *b* = 2.34 (SE = 1.08), *t*(31.00) = −2.17, *p*_adj_ = 0.038. Finally, the feedback timing × outcome valence × ∣Q∣ interaction reached significance, *b* = −11.22 (SE = 3.20), *F*(1, 31.26) = 12.34, *p* = 0.001 (see Figure 5a). Probing this interaction via simple slope analyses indicated that for immediate feedback the FRN/RewP encoded −RPEs, *b* = 12.99 (SE = 2.82), *t*(29.32) = 4.61, *p*_adj_ < 0.001, with no evidence for the encoding of +RPEs (*p*_adj_ = 0.971), whereas no RPE encoding was evident for delayed feedback, that is, neither for +RPEs nor for −RPEs (*p*s_adj_ ≥ 0.741).

**FIGURE 5:**
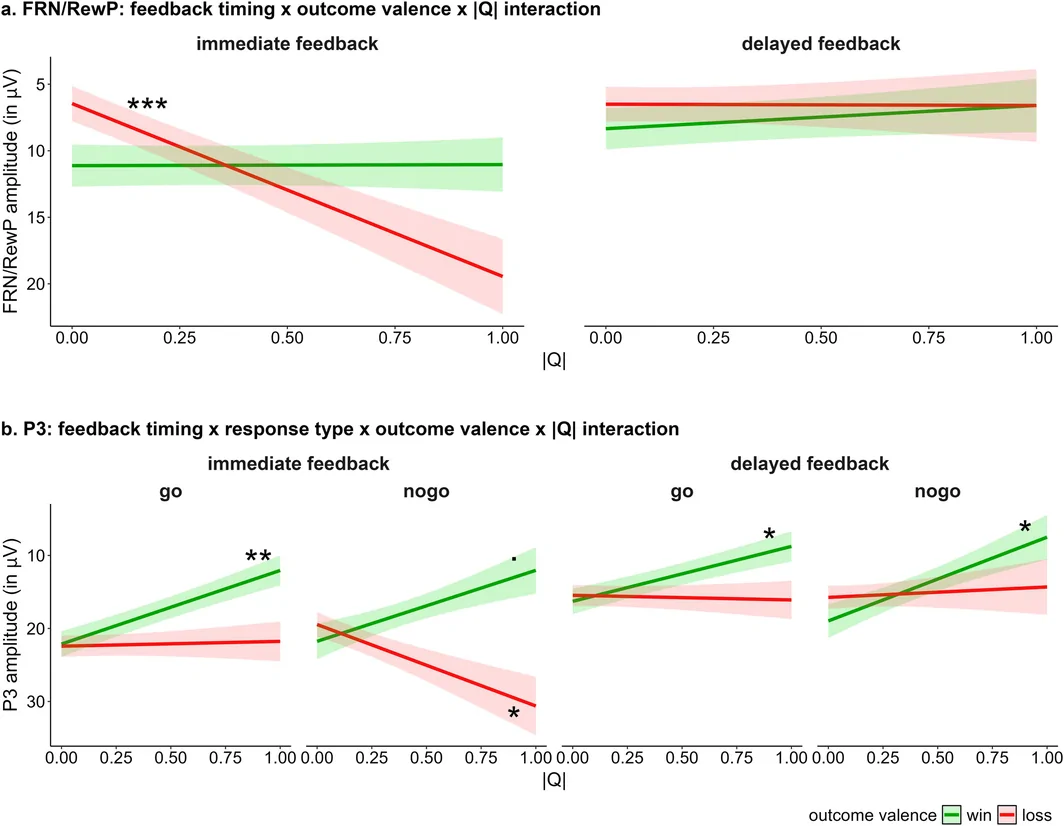
EEG results. (a) Data underlying the significant feedback timing × outcome valence × |*Q*| interaction effect in the FRN/RewP analysis. (b) Descriptive data underlying the significant feedback timing × response type × outcome valence × |*Q*| interaction effect in the P3 analysis. With |*Q*| values close to zero, the presented feedback was unexpected, whereas with |*Q*| values close to one the presented feedback was expected. Data are presented as |*Q*| slopes, as predicted by linear mixed model analyses ± standard error of the mean. Asterisks indicate significant slopes yielded from simple slope analyses. ·< 0.100, *p < 0.050, **p < 0.01, ***p < 0.001.

Again, there were no significant effects involving the predictor response type (*p*s ≥ 0.071; see Table S6). Subordinate exploratory LMM analyses separately for response type replicated the effects reported above for both go (*p*s ≤ 0.014) and nogo choices (*p*s ≤ 0.037). To explore whether RPE encoding was practically equivalent regardless of response type, equivalence testing was performed on the non‐significant response type × outcome valence × ∣Q∣ interaction effect. This revealed that the effect (90% CI = [−3.68, 7.07]) overlapped with but did not entirely lie within the region of practical equivalence ([−0.50, 0.50]), *p* = 0.894; thus, we could not provide evidence for the practical absence of such an effect.

#### 3.3.2 Feedback‐P3

Exploratory analyses for the feedback‐P3 yielded significant main effects of feedback timing, *b* = −6.14 (SE = 2.08), *F*(1, 44.07) = 8.69, *p* = 0.005, and outcome valence, *b* = 4.55 (SE = 0.86), *F*(1, 30.84) = 27.86, *p* < 0.001, indicating generally more positive P3 amplitudes for immediate versus delayed feedback and for *loss* versus *win* feedback. In addition, the main effect of ∣Q∣ was significant, *b* = −3.64 (SE = 1.59), *F*(1, 29.71) = 5.22, *p* = 0.030, suggesting that amplitudes of the P3 were more positive when feedback was more unexpected.

Moreover, results revealed a significant feedback timing × outcome valence interaction effect, *b* = −4.05 (SE = 1.72), *F*(1, 30.84) = 5.51, *p* = 0.025, with the effect of outcome valence being larger for immediate than delayed feedback. Finally, there was a significant outcome valence × ∣Q∣ interaction, *b* = −12.13 (SE = 2.54), *F*(1, 43.50) = 22.89, *p* < 0.001, indicating that the P3 generally encoded a +RPE, *b* = −9.70 (SE = 2.07), *t*(34.02) = −4.69, *p*_adj_ < 0.001, with more positive amplitudes, the more unexpected *win* feedback was, but no evidence for −RPE encoding (*p*_adj_ = 0.235).

Importantly, RPE encoding depended on both the response type and feedback timing, as revealed by a significant response type × outcome valence × ∣Q∣ interaction, *b* = 6.68 (SE = 2.17), *F*(1, 1237.96) = 9.48, *p* = 0.002, that was ultimately qualified by the higher‐order feedback timing × response type × outcome valence × ∣Q∣ interaction effect, *b* = −9.58 (SE = 4.34), *F*(1, 1237.96) = 4.88, *p* = 0.027. Resolving this effect showed that +RPEs were consistently encoded in the P3 across feedback timings and response type (*p*s_adj_ ≤ 0.026, though note a non‐significant trend for nogo with immediate feedback, *p*_adj_ = 0.064). By contrast, the P3 signaled a −RPE only for nogo with immediate feedback, *b* = 11.16 (SE = 4.28), *t*(34.35) = 2.61, *p*_adj_ = 0.027, whereas for all other conditions no such effect was detected (*p*s_adj_ ≥ 0.807; see Figure 5b).

## 4 Discussion

The present study aimed to examine whether feedback timing modulates motivational biases and to test the broader role of (biased) feedback processing in shaping them. To this end, two groups of participants performed a modified orthogonalized go/nogo task, learning from either immediate or delayed feedback. Our results showed that motivational biases governed decision‐making when learning from both immediate and delayed feedback. Yet, results from single‐trial ERP analyses did not provide evidence for biased feedback processing of action over inaction, as predicted by an instrumental learning bias. Together with complementary evidence from computational modeling, our findings therefore do not support biased instrumental learning as a *substantial* mechanism underlying motivational biases.

### 4.1 Motivational Biases in Learning From Immediate and Delayed Feedback

Our results replicate previous findings by showing that participants performed better when learning to execute a response to gain a reward than to avoid punishment, as well as when learning to withhold a response to avoid punishment than to gain a reward (Guitart‐Masip et al. 2014; Guitart‐Masip, Huys, et al. 2012). These motivational biases emerged across both learning from immediate and delayed feedback and were not found to be attenuated for learning from delayed feedback, thereby aligning with evidence showing the robustness of motivational biases to different learning contexts (Ereira et al. 2021; Peterburs et al. 2021; Vahedi et al. 2024). This finding is remarkable, given that motivational biases are argued to rely substantially on the striatum (e.g., de Boer et al. 2019; Guitart‐Masip et al. 2014; Perosa et al. 2020; Richter et al. 2014), which appears to be less strongly involved in learning from delayed feedback (Foerde and Shohamy 2011; Hinneberg and Hegele 2022; Lighthall et al. 2018). Accordingly, motivational biases may be deeply ingrained, not *necessarily* depending on the striatum alone but on a broader network of brain regions. Specifically, previous studies have linked the prefrontal cortex with mediating motivational biases (Algermissen et al. 2024; Cavanagh et al. 2013; Kim et al. 2023; Ousdal et al. 2018). Moreover, the hippocampus, which may be increasingly involved in learning from delayed feedback (Foerde et al. 2013; Foerde and Shohamy 2011), was found to contribute to resolving approach‐avoidance conflicts (Bach et al. 2014; Bryant and Barker 2020; Le Duc et al. 2025; for a review, see Ito and Lee 2016).

Importantly, although striatal recruitment may be reduced under learning from delayed feedback, it may still *contribute* to the emergence of motivational biases. This is further supported by evidence suggesting that processing of immediate and delayed feedback involve at least partially similar mechanisms (Weber and Bellebaum 2024; Weismüller et al. 2018; but also see corresponding evidence from this study, which is discussed below). Hence, our results suggest that motivational biases rely on such fundamental mechanisms that drive learning universally and independent of feedback timing. Taken together, our results therefore corroborate previous findings suggesting motivational biases to constitute a robust hallmark of decision‐making that is only hardly modifiable (e.g., Ereira et al. 2021; Peterburs et al. 2021).

### 4.2 Motivational Biases May Reflect a Pavlovian Response Bias

To gain deeper insight into the computational mechanisms underlying motivational biases, we fitted a series of RL models to the behavioral data (de Boer et al. 2019; Guitart‐Masip, Chowdhury, et al. 2012; Raab and Hartley 2020). Previous work has demonstrated that motivational biases can be entirely (de Boer et al. 2019) or at least partly (Swart et al. 2017) explained by an instrumental learning bias, with facilitated learning from rewarded actions and impaired learning from punished inactions. Our results are not in line with these findings, as model comparison overall favored a model including a Pavlovian—but not instrumental—bias parameter (while all RL models were favored compared with simpler reference models suggesting random responding or a simple win‐stay/lose‐shift policy, see the Supporting Information). This result resonates well with our EEG findings, which did not yield stronger feedback valence coding for action versus inaction, which we had expected may underlie such an instrumental learning bias.

However, it must be acknowledged that most previous studies reporting biased instrumental learning typically employed a designated variant of the orthogonalized go/nogo task, teasing apart more general Pavlovian and action‐selective instrumental effects by introducing separate cues for correct left and right go choices (Algermissen et al. 2024; Swart et al. 2018; Swart et al. 2017; but see de Boer et al. 2019 who employed the standard task). Yet, there still is reasonable cause to doubt biased instrumental learning as a *substantial* mechanism supporting motivational biases, as they have also been shown in observational learning (Peterburs et al. 2021). Specifically, for observational learning, biased instrumental learning—with boosted learning from rewarded actions and weakened learning from punished inactions—cannot be plausibly considered an underlying mechanism, as participants themselves do not engage in go/nogo choices during learning.

Still, this does not mean that biased learning does not contribute to motivational biases at all. Notably, there was a certain degree of heterogeneity in individual model fits, with choice behavior of some participants best explained by biased instrumental learning (see Table S1). Moreover, analysis of choice repetition behavior indicated biased adjustments to feedback. However, whereas this analysis confirmed facilitated learning from rewarded actions, results also suggested facilitated (rather than impaired) learning from punished inactions, thus only partially aligning with the predictions of an instrumental learning bias. Importantly though, it must be noted that such model‐free analysis of biased learning does not allow to disentangle concurrent behavioral mechanisms. As such, increased overall choice repetition of go versus nogo choices likely reflects a general go bias. Moreover, although posterior predictions of the second‐best fitting model, which included an instrumental learning bias, generally captured behavior relatively well (see Supporting Information), it must be emphasized that both average and accumulated model evidence favored a model including a Pavlovian bias and excluding an instrumental learning bias parameter. Taken together, our results therefore suggest that motivational biases were overall *better* explained by a Pavlovian response bias, as proposed in earlier studies (e.g., Guitart‐Masip, Huys, et al. 2012).

### 4.3 Similarities and Differences in Processing Immediate and Delayed Feedback

The FRN/RewP did not show general sensitivity to outcome valence. Note, however, that general encoding of outcome valence with more negative amplitudes for losses than wins was found in an exploratory model‐free analysis, precluding the predictor |*Q*| (see Supporting Information, also see Figure 4). Instead, amplitudes were more negative for losses versus wins only when feedback was unexpected (see, e.g., Weismüller and Bellebaum 2016), while the opposite pattern, that is, more positive amplitudes for losses versus wins, was found when feedback was expected. Such restricted coding of feedback valence may result from win and loss feedback arising through distinct learning contexts (win vs. avoid‐losing), where altered value encoding may particularly affect avoid‐losing contexts (e.g., see Glazer and Nusslock 2022; Kujawa et al. 2013; Novak and Foti 2015; Pfabigan et al. 2015).

More importantly, FRN/RewP results indicated that the FRN/RewP signaled a −RPE for immediate but not delayed feedback. While this finding generally aligns with the idea of a gradual shift away from the striatum with feedback delay (Foerde and Shohamy 2011; Hinneberg and Hegele 2022), it diverges from previous evidence by our group showing that the FRN/RewP reflects a +RPE, independently of feedback timing (Berlijn et al. 2025; Weber and Bellebaum 2024; but see Huvermann et al. 2025). Again, this discrepancy may partly reflect differences in task design, as −RPEs were derived from a loss‐avoidance context and +RPEs from a reward‐seeking context, such that wins and losses never occurred as alternative outcomes for the same cue. Beyond that, we found +RPE encoding across feedback timing conditions for the later P3, consistent with the P3 findings reported in Weber and Bellebaum (2024). Notably, this result pattern—with early sensitivity to −RPEs, and more slowly emerging sensitivity to +RPEs—generally aligns with evidence for a valence‐dependent dissociation of RPE signals during feedback processing (Bai et al. 2015; Hoy et al. 2021). Given their close temporal proximity, these signals may sometimes overlap (Hoy et al. 2021), possibly accounting for the heterogeneity of findings either in favor of the FRN/RewP reflecting a −RPE (e.g., Bai et al. 2015) or +RPE (e.g., Weber and Bellebaum 2024). Ultimately, combined evidence from the present study and earlier work from our group (e.g., Weber and Bellebaum 2024) suggests that particularly +RPE processing may be universally involved in the processing of immediate and delayed feedback, whereas early (‐)RPE processing may be more sensitive to feedback timing and preserved only for processing of immediate feedback. Note, however, that also the P3 encoded a −RPE, that is, selectively for immediate feedback following nogo choices.

### 4.4 Feedback Processing Underlying Motivational Biases

Most importantly, and contrary to our hypotheses, feedback valence coding in the FRN/RewP did not depend on the response type nor did we find any other processing differences in the FRN/RewP for action versus inaction. These findings contrast with prior work reporting stronger feedback valence effects for action than for inaction (Bikute et al. 2022; Di Bernardi Luft et al. 2024; Yeung et al. 2005). Note, however, that in these studies, participants were explicitly instructed to act or not to act, effectively rendering their responses forced. Notably, reduced FRN/RewP valence coding has been typically observed under conditions of reduced agency, such as observational learning (Bellebaum and Colosio 2014; Bellebaum et al. 2010), or with reduced personal responsibility (Li et al. 2010). As individuals typically favor action over inaction (e.g., Sunderrajan and Albarracin 2021; also reflected in a general go bias in this study), instructed actions may naturally carry stronger agency than inactions, potentially explaining the larger feedback valence effects reported in prior studies.

Also, P3 results did not provide evidence for action‐biased feedback processing as predicted by an instrumental learning bias, therefore contrasting with results by Schevernels et al. (2016) (but note that only positive feedback was analyzed in that study). Instead, stronger RPE effects were found following inaction, reflected in selective −RPE coding following nogo choices for immediate feedback. This pattern may indicate that punishments following inaction are processed as more salient, particularly when unexpected, potentially due to difficulties in attributing punishment to inaction (e.g., Swart et al. 2017). Consistent with the role of the P3 in reflecting motivational significance (e.g., San Martín 2012), such increased salience could hence contribute to the finding of stronger −RPE effects for nogo versus go choices. Note, however, that the LMM yielding this result did not support a maximal random‐effects structure (critically lacking the outcome valence × response type × |*Q*|), which may increase the risk of Type I error for this effect and therefore warrants cautious interpretation (see Matuschek et al. 2017). Overall, our results suggest feedback processing to be largely unaffected by the response type, providing no evidence for biased feedback processing for action over inaction.

### 4.5 Limitations and Future Directions

A few limitations must be acknowledged. First, a disadvantage of the task used is that it naturally leads to unequal and, in some cases, limited numbers of trials per condition (e.g., particularly for rewarded nogo choices). Although LMMs are generally robust to imbalanced datasets (Volpert‐Esmond et al. 2018), and examining RPEs may partly mitigate misinterpretation of, in truth, frequency‐induced expectancy effects, future studies should include a larger number of trials for more stable results. Still, it should be noted that unequal trial counts may introduce more general frequency‐related effects on feedback processing (e.g., Krigolson 2018) that may not fully be accounted for by modeling RPEs. Moreover, while both the FRN/RewP and P3 have proven to be well‐established markers of feedback processing (San Martín 2012) shown to reflect RPE‐related activity (Sambrook and Goslin 2015), it may be possible that those measures failed to capture relevant activity underlying biased learning. For instance, biased learning has recently been interpreted in terms of biased credit assignment (Algermissen et al. 2024), which may rely more strongly on brain regions typically not associated with contributing to the FRN/RewP‐P3 complex (see, e.g., Koutsoumpari et al. 2026). Accordingly, studying motivational biases using other ERP components may be worthwhile, with the N170, which is associated with representational reactivation, a potential candidate (see Röhlinger, Albrecht, and Bellebaum 2025; Röhlinger, Albrecht, Ghio, and Bellebaum 2025). Interpreting biased learning as biased credit assignment may have broader implications for studying feedback timing effects, as credit assignment becomes increasingly difficult with temporal delays (Stolyarova 2018). In addition to that, more elaborated and spatially refined imaging techniques may be needed to unveil the neural signatures of biased learning. Note, however, that even our behavioral data did not provide convincing evidence for biased instrumental learning as a substantial mechanism behind motivational biases. Future work is thus needed to clarify its role in motivational biases. Finally, while aiming to control for overall task duration, learning in the immediate feedback group involved an unusually long ITI. Although choice accuracies descriptively matched those from our previous study using short ITIs (Vahedi et al. 2024), we cannot completely rule out that this manipulation induced unintended effects on learning.

## 5 Conclusion

In conclusion, our results replicate robust motivational biases in feedback‐based learning, while showing that the emergence of these biases does not depend on feedback timing. Moreover, results from computational modeling and single‐trial ERP analyses provided no evidence for biased instrumental learning or feedback processing as substantial mechanisms shaping motivational biases. Instead, motivational biases were overall better accounted for by a Pavlovian response bias. Future research is needed to validate these findings and further clarify the neurocognitive mechanisms underlying motivational biases.

## Author Contributions

**Julian Vahedi:** project administration, methodology, formal analysis, data curation, software, visualization, writing – original draft, writing – review and editing, conceptualization. **Sandra Schneider:** investigation, formal analysis, writing – review and editing. **Livia Swiatek:** investigation, writing – review and editing, formal analysis. **Jutta Peterburs:** project administration, methodology, validation, supervision, writing – review and editing. **Christian Bellebaum:** project administration, conceptualization, methodology, validation, resources, supervision, writing – review and editing.

## Funding

Open access funding enabled and organized by Projekt DEAL.

## Ethics Statement

All procedures performed in this study were in accordance with the ethical standards of the institutional and/or national research committee and with the 1964 Helsinki declaration and its later amendments or comparable ethical standards.

## Consent

Informed consent was obtained from all individual participants included in the present study. All individual participants signed informed consent regarding publishing their (anonymized) data.

## Conflicts of Interest

The authors declare no conflicts of interest.

## Supporting information


**Figure S1:** Comparison of posterior predictions from the best fitting model M2 and the second‐best model M3.
**Figure S2:** Descriptive scalp topographies underlying the ERP results.
**Table S1:** Participant‐wise model fits according to the Bayesian information criterion (BIC) with the best fitting model for each participant marked in bold.
**Table S2:** Results from alternative modeling using fixed values of *V*.
**Table S3:** Consistency of modeling results across models using different baseline parameters.
**Table S4:** Generalized linear mixed‐effects model (GLMM) results on choice accuracy.
**Table S5:** Generalized linear mixed‐effects model (GLMM) results on choice repetition.
**Table S6:** Model‐based linear mixed‐effects model (LMM) results on FRN/RewP data.
**Table S7:** Model‐based linear mixed‐effects model (LMM) results on P3 data.
**Table S8:** Model‐free linear mixed‐effects model (LMM) results on FRN/RewP data.
**Table S9:** Model‐free linear mixed‐effects model (LMM) results on P3 data.
**Table S10:** Generalized linear mixed‐effects model (GLMM) results on brain–behavior relationships.
**Table S11:** Generalized linear mixed‐effects model (GLMM) results on brain–behavior relationships.

## Data Availability

Data and code to reproduce all analyses in this manuscript can be found on the Open Science Framework (https://osf.io/27gjw/overview?view_only=1556006846ad42f3bdc326c81f5fd53f). The study was not preregistered.
