# Supplementary material for: Motivational Biases in Go and Nogo Learning From Immediate Versus Delayed Feedback
Source: Eur J Neurosci. 2026 Oct 26;64(8):e70717. doi: 10.1111/ejn.70717 (PMC13710874; doi:10.1111/ejn.70717)
Supplement: Supplementary file 1 [file EJN-64-0-s001.docx]

**Supplement to
Motivational biases in go and nogo learning from immediate versus delayed feedback**

Julian Vahedi^1^, Sandra Schneider^1^, Livia Swiatek^1^, Jutta Peterburs^2^, & Christian Bellebaum^1^

^1^ Heinrich Heine University Düsseldorf, Düsseldorf, Germany, Faculty of Mathematics and Natural Sciences

^2^ MSH Medical School Hamburg, Germany, Institute for Systems Medicine and Department of Human Medicine

Address for correspondence:

Julian Vahedi, M.Sc. Psych.

Heinrich Heine University Düsseldorf

Institute for Experimental Psychology, Department of Biological Psychology

Universitätsstr. 1, D-40225 Düsseldorf, Germany

E-Mail: [Julian.Vahedi@hhu.de](mailto:Julian.Vahedi@hhu.de)

# Computational modeling of behavioral data

## Modeling of a value-decay during awaiting of feedback

After the main modeling revealed M2 with a go bias parameter $b$ a Pavlovian bias parameter $\pi$, and an inverse temperature parameter $\beta$ (see main manuscript), we also tested the possibility of trial-level value decay (Collins, 2018; Kato & Morita, 2016). Specifically, we assumed that particularly during learning from delayed feedback, the temporal gap between (in)action and feedback—with increased demands in attentional binding and working memory maintenance—may lead to decaying reward expectancies. Therefore, in an exploratory M9, we tested whether inclusion of a decay parameter $\varphi\in[0,1]$ improved model fit compared to the winning model M2. Trial-level value decay was implemented before conventional RPE-based $Q$-value updating with $\varphi$ serving as an *unlearning* rate

$$Q_{t}\left( a_{t},s_{t} \right)= Q_{t-1}\left( a_{t},s_{t} \right)+ \varphi(Q_{0}-Q_{t-1}\left( a_{t},s_{t} \right))$$

Accordingly, $Q$-values were allowed to decay towards the initial $Q$-value ($Q_{0}$=0), where values of $\varphi$ close to 0 would indicate no or little unlearning and values of $\varphi$ close to 1 high unlearning speed. Crucially, model comparison between M2 and M9 suggested that M2 was favorable over M9 (ΔBIC_mean_ = 5.06).

## Alternative modelling using fixed values of $\boldsymbol{V}$

Importantly, most previous studies reporting an instrumental learning bias used fixed rather than learned values of $V$ (+0.5 for win cues, -0.5 for avoid-losing cues) with corresponding initialization values of ± 0.5 for $Q$, upon first reveal of a non-neutral outcome in their implementation of the Pavlovian response bias (e.g., Swart et al., 2017; but see de Boer et al., 2019). This is usually done when cue valence was instructed, and participants are expected to re-adjust their reward expectancies for a given cue once their valence was revealed by non-neutral outcomes. Although cue valence was not explicitly instructed in this study, we exploratively examined whether a model assuming fixed values of $V$would be able to better disentangle the Pavlovian response and instrumental learning biases. Re-running the models according to this approach reproduced the results from the main manuscript, i.e., favoring M2 including a Pavlovian rather than instrumental learning bias parameter, but with overall lower model fit as indicated by the BIC (see Table S1).

## Consistency across of modelling results across different baseline parameters

Given heterogeneity in previous literature concerning the inclusion of parameters that control the balance between exploration and exploitation, we explored the consistency of our modelling results depending on such baseline parameters. Therefore, we re-ran models M0-M8 with alternative parameters controlling the exploration-exploitation tradeoff rather than the inverse temperature parameter $\beta$. First, modelling was performed without using any parameter to account for exploration/exploitation, such that higher values of $W_{t}\left( a_{t},s_{t} \right)$ deterministically led to higher choice probabilities,

$$p(a_{t}| s_{t})= \frac{\exp\left( W_{t}\left( a_{t},s_{t} \right) \right)}{\sum_{a^{'}} {exp(W}_{t}\left( a^{'},s_{t} \right))}$$

Second, instead of an inverse temperature parameter, we used an irreducible noise or lapse rate parameter $\xi\in(0, 1)$, capturing slips of attention by ignoring the learned values entirely if $\xi$ equals 1 (see e.g.,Guitart-Masip et al., 2012; Raab & Hartley, 2020):

$$p(a_{t}| s_{t})= \frac{\exp\left( W_{t}\left( a_{t},s_{t} \right) \right)}{\sum_{a^{'}} {exp(W}_{t}\left( a^{'},s_{t} \right))} (1-\xi)+ \frac{\xi}{2}$$

Third, we used a standard softmax function without $\beta$ or $\xi$, but controlled the exploration/exploitation tradeoff during learning rather than at the decision stage, via a feedback sensitivity parameter $\rho\in(0, +\infty)$:

$$Q_{t}\left( a_{t},s_{t} \right)= Q_{t-1}\left( a_{t},s_{t} \right)+ \varepsilon(\rho r_{t}-Q_{t-1}\left( a_{t},s_{t} \right))$$

Finally, we allowed separate feedback sensitivity parameters for win ${(\rho}^{+})$ and loss${(\rho}^{-})$ outcomes, i.e.:

$$Q_{t}\left( a_{t},s_{t} \right)= Q_{t-1}\left( a_{t},s_{t} \right)+ \varepsilon(\rho^{+/-}r_{t}-Q_{t-1}\left( a_{t},s_{t} \right))$$

Crucially, regardless of which baseline parameters were used, M2 was the best-fitting model. Notably, modelling M2 using $\rho^{(+/-)}$ resulted in a fairly comparable model fit (ΔBIC_mean_ ≤ 0.90), whereas all the other models provided an increasingly worse fit (ΔBIC_mean_ ≥ 14.44; see Table S2).

## Random decision-making versus win-stay/lose-shift strategy?

While the RL models reported in the main manuscript assume quite sophisticated interactions between Pavlovian and instrumental mechanisms underlying choice behavior, we additionally examined whether choice behavior could be captured just as well by simpler decision strategies. We therefore first fitted a random model that assigned equal probability to go and nogo choices on every single trial throughout the whole experiment, effectively reflecting random decision-making without any learning involved. Second, we fitted a noisy win-stay-lose-shift RL model (see Wilson & Collins, 2019). This model assumed that, for a given cue, participants repeat rewarded choices and shift away from punished choices, while the free parameter $\vartheta$ $\in[0, 1]$ reflected overall randomness in decision-making. Importantly, we assumed this win-stay/lose-shift strategy should only apply to non-neutral feedback, hence following neutral feedback we assumed random decision-making. Accordingly, choice probabilities $p\left( a_{t}| s_{t} \right)$ were calculated as follows:

$$p\left( a_{t}| s_{t} \right)= \begin{matrix} 0.5 \\ 1- \\ \vartheta/2 \end{matrix} \vartheta/2\begin{matrix} \\ \\ \end{matrix}\begin{matrix} if neutral feedback \\ if action followed win-stay/lose-shift rule \\ otherwise \end{matrix}$$

While model comparison suggested that mean BIC values were lower for the win-stay/lose shift model (mean BIC =199.56) compared to the random model (mean BIC = 221.81), both models showed larger BIC values compared to the best-fitting model M2 (mean BIC = 161.20) and were in fact outperformed by every other RL model reported in the main manuscript.

These findings indicate that participants’ choice behavior is better explained by RL models than by either random responding or a simple win-stay/lose-shift strategy.

# Supplementary exploration of relationships between BIS/BAS and motivational biases

To explore relationships between BIS/BAS scores and motivational biases, we performed Spearman rank-order correlation analysis between participant-wise random slopes for the cue valence × required response interaction effect and BIS and BAS scores, following Vahedi et al. (2024). In line with the results reported in Vahedi et al. (2024), there was no significant relationship between BIS/BAS scores and the expression of motivational biases (*ps* ≥ .322).

# Supplementary P3 analyses

In addition to analyzing the P3 at a centro-parietal electrode cluster (Cz, CP1, CP2, Pz), we re-ran analogous analyses at a fronto-central cluster (Fz, FC1, FC2, FCz), to account for the distinction between fronto-central P3a and centro-parietal P3b (Polich, 2007; Polich & Criado, 2006). For completeness, P3 scoring at the fronto-central cluster produced slightly different extraction windows: 319–379 ms for the immediate feedback group and 349–409 ms for the delayed feedback group. Results mirrored those from the centro-parietal cluster and are summarized in Tables S6 and S8.

# Supplementary model-free analyses

We additionally performed model-free analyses, by re-running the LMMs, described in the main manuscript, but excluding the predictor |Q|. Results confirmed the well-established coding of valence in the FRN/RewP, as reflected by a significant main effect of outcome valence, with more negative amplitudes for losses compared to wins, *b* = -1.35 (*SE* = 0.52), *F*(1, 46.17) = 6.70, *p* = .013. Notably, apart from that, no other effects reached statistical significance (*p*s ≥ .081; see Table S3). Given the theoretical importance of the response type × outcome valence interaction, which was assumed to underly an instrumental learning bias, practical absence of this effect was examined via equivalence testing. The effect (90% CI = [-2.20, 0.04]) overlapped with, but did not lie entirely within the region of practical equivalence ([-0.50, 0.50]), *p* = .814; thus, we could not provide evidence for the practical absence of this effect.

More exploratory analyses of the feedback-P3 again revealed a significant main effect of outcome valence, with more positive amplitudes for unfavorable compared to favorable feedback, *b* = 2.38 (*SE* = 0.76), *F*(1, 46.94) = 9.83, *p* = .003. Moreover, a significant main effect was also found for feedback timing, *b* = -4.56 (*SE* = 1.79), *F*(1, 51.28) = 6.52, *p* = .014, with overall more positive amplitudes in the immediate versus delayed feedback group. Importantly, the significant feedback timing × outcome valence interaction effect, *b* = -3.46 (*SE* = 1.52), *F*(1, 46.94) = 5.18, *p* = .027, indicated an effect of outcome valence emerging only for immediate, *b* = 4.11 (*SE* = 1.10), *t*(48.78) = 3.74, *p_adj_* = .001, but not for delayed feedback (*p_adj_* = .537). Again, the response type × outcome valence interaction was non-significant (*p* = .281). Conducting equivalence testing for this effect once more revealed that the 90% CI ([-3.08, 0.62]) overlapped with, but did not entirely lie within the region of practical equivalence ([-0.50, 0.50]), *p* = .804, thereby failing to provide evidence for the practical absence of this effect.

# Exploratory investigation of brain-behavior relationships

As model-based EEG analyses suggested that the FRN/RewP encodes -RPEs (at least in the immediate feedback group) and the P3 encodes +RPEs (see main manuscript), we directly explored brain-behavior relationships. Specifically, if -RPEs (or loss feedback) facilitate future nogo choices, and +RPEs (or win feedback) facilitate future go choices (see Collins & Frank, 2014; Frank, 2005; Frank et al., 2004), then trial-by-trial FRN/RewP and/or P3 amplitudes may predict the next-trial choice for the same cue. Accordingly, we specified separate generalized linear mixed-effects models (GLMM) including feedback timing (*immediate [=reference], delayed*) and outcome valence (*win [=reference], loss*) as categorical predictors and amplitudes of either the FRN/RewP, or P3 as a continuous predictor. Because our primary interest was in within-subject variation, FRN/RewP and P3 amplitudes were centered on each participant’s mean and scaled (divided by 10) prior to model fitting (LoTemplio et al., 2023; also see Enders & Tofighi, 2007). The choice on the next trial for the same cue was set as the dependent variable (0/1 = nogo/go). Detailed results tables are provided in Table S9 and Table S10.

The FRN/RewP model revealed a significant main effect of outcome valence, *b* = -1.16 (*SE* = 0.20), *χ^2^*(1) = 34.36, *p* < .001, indicating that win compared to loss feedback led to more go choices on the next trial. Crucially, there was no significant effect involving the predictor FRN/RewP amplitude (*p*s ≥ .054). The P3 model again revealed the significant main effect of outcome valence, *b* = -1.15 (*SE* = 0.20), *χ^2^*(1) = 33.63, *p* < .001. Moreover, we found a significant main effect of P3 amplitude, *b* = -0.06 (*SE* = 0.03), *χ^2^*(1) = 4.34, *p* = .037, indicating that a more negative P3 predicted a higher go tendency on the next trial. Results were similar when scoring the P3 over fronto-central electrodes (see Table S10).

# References

Collins, A. G. (2018). The tortoise and the hare: Interactions between reinforcement learning and working memory. *Journal of Cognitive Neuroscience, 30*(10), 1422-1432.

Collins, A. G., & Frank, M. J. (2014). Opponent actor learning (OpAL): modeling interactive effects of striatal dopamine on reinforcement learning and choice incentive. *Psychological Review, 121*(3), 337.

de Boer, L., Axelsson, J., Chowdhury, R., Riklund, K., Dolan, R. J., Nyberg, L., Bäckman, L., & Guitart-Masip, M. (2019). Dorsal striatal dopamine D1 receptor availability predicts an instrumental bias in action learning. *Proceedings of the National Academy of Sciences, 116*(1), 261-270.

Enders, C. K., & Tofighi, D. (2007). Centering predictor variables in cross-sectional multilevel models: a new look at an old issue. *Psychological Methods 12*(2), 121.

Frank, M. J. (2005). Dynamic dopamine modulation in the basal ganglia: a neurocomputational account of cognitive deficits in medicated and nonmedicated Parkinsonism. *Journal of Cognitive Neuroscience, 17*(1), 51-72.

Frank, M. J., Seeberger, L. C., & O'reilly, R. C. (2004). By carrot or by stick: cognitive reinforcement learning in parkinsonism. *Science, 306*(5703), 1940-1943.

Guitart-Masip, M., Huys, Q. J., Fuentemilla, L., Dayan, P., Duzel, E., & Dolan, R. J. (2012). Go and no-go learning in reward and punishment: interactions between affect and effect. *Neuroimage, 62*(1), 154-166.

Kato, A., & Morita, K. (2016). Forgetting in reinforcement learning links sustained dopamine signals to motivation. *PLoS Computational Biology, 12*(10), e1005145.

LoTemplio, S. B., Lopes, C. L., McDonnell, A. S., Scott, E. E., Payne, B. R., & Strayer, D. L. (2023). Updating the relationship of the Ne/ERN to task-related behavior: A brief review and suggestions for future research. *Frontiers in Human Neuroscience 17*, 1150244.

Polich, J. (2007). Updating P300: an integrative theory of P3a and P3b. *Clinical Neurophysiology, 118*(10), 2128-2148.

Polich, J., & Criado, J. R. (2006). Neuropsychology and neuropharmacology of P3a and P3b. *International Journal of Psychophysiology, 60*(2), 172-185.

Raab, H. A., & Hartley, C. A. (2020). Adolescents exhibit reduced Pavlovian biases on instrumental learning. *Scientific Reports, 10*(1), 15770.

Swart, J. C., Froböse, M. I., Cook, J. L., Geurts, D. E., Frank, M. J., Cools, R., & Den Ouden, H. E. (2017). Catecholaminergic challenge uncovers distinct Pavlovian and instrumental mechanisms of motivated (in) action. *Elife, 6*, e22169.

Vahedi, J., Mundorf, A., Bellebaum, C., & Peterburs, J. (2024). Emotional cues reduce Pavlovian interference in feedback-based go and nogo learning. *Psychological Research*, 1-19.

Wilson, R. C., & Collins, A. G. (2019). Ten simple rules for the computational modeling of behavioral data. *Elife, 8*, e49547.

# Figure S1

*Note*: Comparison of posterior predictions from the best-fitting model M2 and the second-best model M3. Model parameters: $\varepsilon$ = learning rate; $b$ = go bias parameter; $\pi$ = Pavlovian bias parameter; $k$ = instrumental learning bias parameter; $\beta$ = inverse temperature parameter. Abbreviations: BIC = Bayesian information criterion; GW = go to win; GAL = go to avoid-losing; NGW = nogo to win; NGAL = nogo to avoid-losing.

# Figure S2

*Note*: Descriptive scalp topographies underlying the ERP results. Data represent the difference between unexpected (|Q| ≤ 0.$\bar{3}$) and expected feedback (|Q| ≥ 0.$\bar{6}$), i.e., unexpected-expected, where **a.** shows scalp topographies within 217-277 ms (immediate feedback) and 242-302 ms (delayed feedback), corresponding to the FRN/RewP results and **b.** shows scalp topographies within 325-385 ms (immediate feedback) and 335-395 ms (delayed feedback), corresponding to the P3 results. Abbreviations: +RPE = positive reward prediction error; -RPE = negative reward prediction error.

# Table S1

| participant | feedback timing | M0 | M1 | M2 | M3 | M4 | M5 | M6 | M7 | M8 |
| --- | --- | --- | --- | --- | --- | --- | --- | --- | --- | --- |
| vp01 | immediate | **84.61** | 88.00 | 93.01 | 93.19 | 92.69 | 92.95 | 97.87 | 97.76 | 97.09 |
| vp02 | immediate | 194.96 | 194.45 | **175.78** | 186.47 | 184.57 | 189.81 | 176.02 | 181.18 | 181.66 |
| vp03 | delayed | 203.74 | 205.74 | **193.18** | 198.12 | 210.01 | 199.65 | 198.26 | 198.20 | 198.12 |
| vp04 | immediate | 170.18 | 174.02 | 103.61 | 103.84 | 148.24 | **103.60** | 109.41 | 110.83 | 109.74 |
| vp06 | delayed | 215.43 | 216.67 | **202.13** | 220.99 | 208.68 | 222.27 | 211.51 | 210.96 | 211.75 |
| vp07 | delayed | 184.73 | 184.96 | 176.71 | **176.59** | 180.02 | 179.72 | 181.52 | 181.77 | 182.32 |
| vp08 | delayed | 145.90 | **139.14** | 143.60 | 147.34 | 147.10 | 147.07 | 147.17 | 150.08 | 149.97 |
| vp09 | delayed | **216.46** | 221.29 | 226.27 | 226.32 | 226.35 | 226.32 | 227.10 | 231.32 | 231.26 |
| vp10 | immediate | 72.43 | 54.52 | **47.19** | 50.76 | 59.71 | 50.79 | 51.98 | 52.45 | 51.98 |
| vp12 | delayed | **81.20** | 81.80 | 86.82 | 86.86 | 86.78 | 86.86 | 91.87 | 91.82 | 91.78 |
| vp13 | delayed | 191.79 | **153.41** | 154.01 | 158.25 | 161.69 | 157.86 | 162.63 | 162.32 | 161.84 |
| vp14 | immediate | 153.78 | **99.82** | 102.69 | 104.62 | 103.82 | 105.74 | 108.91 | 107.99 | 109.05 |
| vp15 | delayed | 186.00 | 187.70 | 171.79 | **170.99** | 183.59 | 173.95 | 175.32 | 176.98 | 174.16 |
| vp17 | delayed | 227.21 | **210.06** | 213.06 | 219.85 | 220.33 | 220.35 | 223.84 | 223.84 | 223.83 |
| vp18 | immediate | 163.35 | 148.48 | 138.80 | 143.85 | 149.97 | 145.94 | **122.72** | 143.77 | 143.79 |
| vp19 | immediate | 191.38 | **161.92** | 165.98 | 169.94 | 165.94 | 170.43 | 173.23 | 172.40 | 172.56 |
| vp20 | immediate | **89.97** | 91.19 | 93.31 | 96.00 | 97.87 | 96.16 | 100.82 | 101.09 | 100.90 |
| vp21 | delayed | 191.55 | 168.10 | 163.12 | 167.04 | **161.88** | 170.23 | 167.99 | 168.04 | 168.68 |
| vp23 | immediate | **224.82** | 225.22 | 229.82 | 231.39 | 231.39 | 231.39 | 236.15 | 236.16 | 236.16 |
| vp24 | delayed | 133.35 | 138.08 | 135.93 | **126.68** | 137.69 | 126.92 | 131.37 | 141.60 | 131.94 |
| vp25 | delayed | 195.61 | 166.65 | 166.56 | 170.12 | **165.99** | 172.57 | 171.58 | 170.71 | 171.54 |
| vp26 | delayed | 158.00 | 162.20 | 95.99 | **95.24** | 140.53 | 95.86 | 101.40 | 103.60 | 102.12 |
| vp27 | immediate | 179.03 | **159.78** | 164.85 | 164.35 | 165.60 | 164.41 | 169.68 | 170.92 | 169.76 |
| vp28 | immediate | 193.35 | 172.37 | **161.41** | 166.43 | 166.13 | 169.58 | 165.39 | 166.46 | 165.78 |
| vp29 | immediate | 185.17 | 147.17 | 147.25 | 150.93 | **144.22** | 153.51 | 153.13 | 150.53 | 152.41 |
| vp30 | immediate | 216.53 | **203.95** | 208.93 | 211.97 | 212.04 | 212.00 | 215.57 | 215.57 | 215.56 |
| vp31 | immediate | 191.05 | 187.57 | **186.24** | 189.94 | 190.06 | 197.04 | 193.96 | 192.95 | 193.53 |
| vp32 | delayed | 181.21 | **164.47** | 166.43 | 169.23 | 169.55 | 169.78 | 172.64 | 172.76 | 172.62 |
| vp33 | delayed | 212.94 | 209.65 | **201.16** | 214.81 | 211.22 | 220.56 | 207.38 | 209.76 | 206.68 |
| vp34 | delayed | 95.25 | **93.80** | 98.75 | 99.94 | 99.38 | 99.95 | 104.36 | 104.07 | 104.81 |
| vp35 | delayed | 196.42 | 192.75 | **178.28** | 185.96 | 189.84 | 187.12 | 187.74 | 187.37 | 187.63 |
| vp36 | delayed | 119.54 | 124.31 | 121.72 | 111.94 | 127.66 | **111.40** | 115.24 | 130.35 | 115.35 |
| vp38 | immediate | **159.26** | 164.32 | 169.19 | 169.52 | 170.87 | 169.65 | 168.39 | 174.21 | 169.21 |
| vp39 | immediate | 92.54 | **91.52** | 95.50 | 96.10 | 96.48 | 95.42 | 100.61 | 100.30 | 100.58 |
| vp41 | immediate | 189.50 | 189.90 | **171.77** | 178.86 | 175.34 | 187.44 | 179.28 | 178.14 | 178.12 |
| vp42 | delayed | 213.68 | 210.85 | **207.85** | 208.52 | 211.15 | 215.55 | 211.73 | 211.58 | 213.45 |
| vp43 | immediate | 192.00 | 187.23 | **182.48** | 189.19 | 192.66 | 193.17 | 188.37 | 188.21 | 188.32 |
| vp44 | immediate | 195.54 | 177.56 | 181.20 | 184.64 | 183.40 | 184.49 | **173.41** | 182.69 | 187.56 |
| vp45 | immediate | 167.34 | 161.39 | 157.52 | 163.81 | 166.15 | 163.15 | **152.99** | 162.55 | 164.29 |
| vp46 | immediate | 80.94 | 86.15 | 83.93 | **80.17** | 89.48 | 80.64 | 86.35 | 88.88 | 87.20 |
| vp47 | delayed | **203.82** | 207.90 | 212.46 | 215.75 | 215.79 | 215.74 | 220.33 | 220.32 | 220.31 |
| vp48 | delayed | **221.27** | 223.06 | 228.11 | 227.99 | 227.97 | 228.12 | 232.98 | 233.02 | 233.17 |
| vp49 | immediate | 189.24 | 181.74 | **173.08** | 174.95 | 173.29 | 178.03 | 178.17 | 177.44 | 178.88 |
| vp50 | immediate | 221.58 | **211.33** | 214.90 | 216.62 | 216.69 | 216.61 | 221.19 | 221.53 | 221.30 |
| vp51 | delayed | 134.50 | 133.85 | 138.76 | 137.02 | 141.89 | 134.52 | 133.81 | 146.25 | **132.03** |
| vp52 | immediate | **222.05** | 225.72 | 230.47 | 230.76 | 230.78 | 230.77 | 235.58 | 235.58 | 235.58 |
| vp53 | immediate | **184.94** | 185.22 | 188.64 | 190.94 | 188.98 | 191.89 | 187.88 | 189.80 | 190.94 |
| vp54 | delayed | 215.41 | **193.54** | 198.54 | 202.35 | 202.36 | 202.38 | 207.33 | 207.33 | 207.35 |
| vp55 | delayed | 135.71 | 131.93 | 132.35 | 124.52 | **124.49** | 130.26 | 125.96 | 129.75 | 135.54 |
| vp56 | delayed | 189.91 | **166.87** | 167.34 | 168.17 | 172.44 | 169.45 | 171.85 | 172.37 | 172.26 |
| vp57 | immediate | **124.26** | 128.78 | 133.78 | 135.54 | 135.58 | 135.62 | 140.05 | 140.63 | 140.43 |
| vp58 | immediate | **83.66** | 88.72 | 93.68 | 94.33 | 93.05 | 94.59 | 97.77 | 97.03 | 99.19 |
| vp59 | delayed | 207.82 | **192.50** | 193.04 | 198.42 | 198.99 | 199.47 | 201.26 | 201.22 | 201.31 |
| vp60 | delayed | 208.37 | **208.10** | 208.62 | 214.09 | 214.13 | 214.11 | 215.79 | 215.84 | 215.83 |
| vp61 | immediate | 219.29 | **209.56** | 214.48 | 217.97 | 218.12 | 218.12 | 222.97 | 223.14 | 223.15 |
| vp62 | delayed | 164.07 | 153.57 | **135.28** | 139.90 | 138.38 | 146.00 | 143.08 | 141.70 | 142.66 |
| *Note*: Participant-wise model fits according to the Bayesian Information Criterion (BIC) with the best-fitting model for each participant marked in bold. | | | | | | | | | | |

# Table S2

|  | mean.BIC | std.BIC |
| --- | --- | --- |
| M0: ε + β | 190.46 | 39.78 |
| M1: ε + b + β | 175.78 | 38.27 |
| **M2: ε + b + π + β** | **163.48** | **44.42** |
| M3: ε + b + k + β | 174.70 | 41.34 |
| M4: ε + b + k_rewGo_ + β | 171.44 | 42.96 |
| M5: ε + b + k_punNogo_ + β | 177.09 | 41.08 |
| M6: ε + b + k + π + β | 167.85 | 45.34 |
| M7: ε + b + k_rewGo_ + π + β | 168.73 | 45.19 |
| M8: ε + b + k_punNogo_ + π + β | 169.01 | 45.22 |
| *Note*: Results from alternative modelling using fixed values of V. Consistent with the modelling approach described in the main manuscript, using fixed values of V (±0.5) with corresponding initialization values of ±0.5 for Q, after the first non-neutral outcome for a given cue, modelling results revealed M2 as the best-fitting model. Model parameters: $\varepsilon$ = learning rate; $b$ = go bias parameter; $\pi$ = Pavlovian bias parameter; $k$ = instrumental learning bias parameter; $\beta$ = inverse temperature parameter. Abbreviations: BIC = Bayesian information criterion | | |

# Table S3

|  | no baseline param | ξ | ρ | ρ^+^ + ρ^-^ |
| --- | --- | --- | --- | --- |
| M0 | 190.42 (21.34) | 195.46 (21.14) | 172.56 (44.56) | 171.78 (45.85) |
| M1 | 180.59 (22.97) | 185.63 (22.71) | 165.06 (43.63) | 165.11 (44.69) |
| **M2** | **175.64 (27.24)** | **180.70 (27.06)** | **161.36 (44.26)** | **162.10 (44.46)** |
| M3 | 183.83 (23.64) | 188.78 (23.25) | 163.87 (45.90) | 165.00 (46.21) |
| M4 | 185.08 (23.34) | 190.18 (23.11) | 166.42 (43.72) | 166.95 (44.50) |
| M5 | 184.90 (23.56) | 189.80 (23.12) | 165.19 (46.22) | 166.28 (46.51) |
| M6 | 181.15 (27.43) | 186.26 (27.32) | 165.37 (45.26) | 167.90 (45.23) |
| M7 | 181.45 (27.80) | 186.36 (27.40) | 167.08 (44.51) | 168.09 (44.94) |
| M8 | 181.54 (27.81) | 186.38 (27.32) | 166.54 (44.96) | 167.94 (45.23) |
| *Note*: Consistency of modelling results across models using different baseline parameters. Data are presented as mean BIC with standard deviation in brackets. Across all models, M2 (including a go bias and Pavlovian response bias parameter) was the best-fitting model. Baseline parameters: β= inverse temperature parameter; ξ = irreducible noise/lapse rate; ρ^(+/-)^ = feedback sensitivity parameter (for wins and losses). Abbreviations: BIC = Bayesian information criterion; param = parameter. | | | | |

# Table S4

| *Generalized Linear Mixed-Effects Model (GLMM) Results on Choice Accuracy* | | | | | |
| --- | --- | --- | --- | --- | --- |
| *Model formula* |  |  |  |  |  |
|  | Accuracy ~ 1 + Feedback Timing + Cue Valence + Feedback Timing:Cue Valence + Required Response + Feedback Timing:Required Response + Cue Valence:Required Response + Feedback Timing:Cue Valence:Required Response +  (1 + Cue Valence + Required Response + Cue Valence:Required Response \| Participant) | | | | |
| *Sampling units* |  |  |  |  |  |
|  | N observations = 8655 | | | | |
|  | N participants = 56 (28 immediate, 28 delayed feedback) | | | | |
|  |  |  |  |  |  |
| *Fixed effects* |  |  |  |  |  |
|  | *b* | *SE* | *χ2* | *df* | *p* |
| **Intercept** | **0.79** | **0.10** | 72.77 | 1 | **<.001** |
| Feedback Timing | -0.21 | 0.21 | 1.03 | 1 | .311 |
| **Cue Valence** | **-0.37** | **0.15** | **5.76** | **1** | **.016** |
| **Required Response** | **-1.8** | **0.17** | **109.16** | **1** | **<.001** |
| Feedback Timing:Cue Valence | -0.24 | 0.3 | 0.63 | 1 | .426 |
| Feedback Timing:Required Response | 0.09 | 0.33 | 0.07 | 1 | .788 |
| **Cue Valence:Required Response** | **2.96** | **0.39** | **57.25** | **1** | **<.001** |
| Feedback Timing:Cue Valence:Required Response | -0.3 | 0.76 | 0.15 | 1 | .695 |
|  |  |  |  |  |  |
| *Random effects* |  |  |  |  |  |
|  | Variance | *SD* | Correlation | | |
| Participant (Intercept) | 0.57 | 0.75 |  | | |
| Participant (Cue Valence) | 0.98 | 0.99 | -0.72 | | |
| Participant (Required Response) | 1.32 | 1.15 | 0.13 -0.41 | | |
| Participant (Cue Valence:Required Response) | 7.23 | 2.69 | 0.05 0.26 -0.92 | | |
|  |  |  |  |  |  |
| *Goodness of fit* |  |  |  |  |  |
|  | AIC | BIC | LL | Deviance | |
|  | 8864.3 | 8991.5 | -4414.2 | 8828.3 | |
|  | Marginal R^2^ | |  | Conditional R^2^ | |
|  | 0.224 | |  | 0.478 | |
| *Note:* Abbreviations: SE = standard error, SD = standard deviation; AIC = Akaike information criterion; BIC = Bayesian information criterion; LL = log-likelihood. Significant effects are highlighted in bold. | | | | | |

# Table S5

| *Generalized Linear Mixed-Effects Model (GLMM) Results on Choice Repetition* | | | | | | | |
| --- | --- | --- | --- | --- | --- | --- | --- |
| *Model formula* |  | | | | | | |
|  | Choice Repetition ~ 1 + Feedback Timing + Response Type + Feedback Timing:Response Type + Outcome Valence + Feedback Timing:Outcome Valence + Response Type:Outcome Valence + Feedback Timing:Response Type:Outcome Valence + (1 + Outcome Valence + Response Type \| Participant) | | | | | | |
|  |  | | | | | | |
| *Sampling units* |  | | | | | | |
|  | N observations = 3820 | | | | | | |
|  | N participants = 56 (28 immediate, 28 delayed feedback) | | | | | | |
|  |  | | | | | | |
| *Fixed effects* |  | | | | | | |
|  | *b* | *SE* | | *χ2* | | *df* | *p* |
| Intercept | 0.83 | 0.12 | | 48.09 | | 1 | <.001 |
| Feedback Timing | -0.25 | 0.23 | | 1.14 | | 1 | .285 |
| Response Type | -1.12 | 0.12 | | 88.84 | | 1 | <.001 |
| Outcome Valence | -2.17 | 0.23 | | 92.22 | | 1 | <.001 |
| Feedback Timing:Response Type | -0.17 | 0.23 | | 0.55 | | 1 | .459 |
| Feedback Timing:Outcome Valence | 0.65 | 0.44 | | 2.13 | | 1 | .145 |
| Response Type:Outcome Valence | 1.42 | 0.19 | | 55.22 | | 1 | <.001 |
| Feedback Timing:Response Type:Outcome Valence | -0.29 | 0.37 | | 0.63 | | 1 | .429 |
|  |  | | | | | | |
| *Random effects* |  | | | | | | |
|  | Variance | *SD* | | Correlation | | | |
| Participant (Intercept) | 0.62 | 0.79 | |  | | | |
| Participant (Outcome Valence) | 2.16 | 1.47 | | 0.94 | | | |
| Participant (Response Type) | 0.27 | 0.52 | | 0.35 0.50 | | | |
|  |  | | | | | | |
| *Goodness of fit* |  | | | | | | |
|  | AIC | | BIC | | LL | Deviance | |
|  | 3679.3 | | 3766.8 | | -1825.7 | 3651.3 | |
|  | Marginal R^2^ | | | | Conditional R^2^ | | |
|  | 0.306 | | | | 0.505 | | |
| *Note:* Abbreviations: SE = standard error, SD = standard deviation; AIC = Akaike information criterion; BIC = Bayesian information criterion; LL = log-likelihood. Significant effects are highlighted in bold. | | | | | | | |

# Table S6

| *Model-Based Linear Mixed-Effects Model (LMM) Results on FRN/RewP Data* | | | | | | | | |
| --- | --- | --- | --- | --- | --- | --- | --- | --- |
| *Model formula* |  | | | | | | | |
|  | FRN/RewP ~ 1 + Feedback Timing + Response Type + Feedback Timing:Response Type + Outcome Valence + Feedback Timing:Outcome Valence + Response Type:Outcome Valence + Feedback Timing:Response Type:Outcome Valence + \|Q\| + Feedback Timing:\|Q\| + Response Type:\|Q\| + Feedback Timing:Response Type:\|Q\| + Outcome Valence:\|Q\| + Feedback Timing:Outcome Valence:\|Q\| + Response Type:Outcome Valence:\|Q\| + Feedback Timing:Response Type:Outcome Valence:\|Q\| + (1 + Outcome Valence + \|Q\| + Outcome Valence:\|Q\| + Response Type + Response Type:\|Q\| + Response Type:Outcome Valence + Response Type:Outcome Valence:\|Q\| \| Participant) + (1 \| Electrode) | | | | | | | |
|  |  | | | | | | | |
| *Sampling units* |  | | | | | | | |
|  | N observations = 16452 | | | | | | | |
|  | N participants = 56 (28 immediate, 28 delayed feedback) | | | | | | | |
|  | N electrodes = 4 (Fz, FCz, FC1, FC2) | | | | | | |  |
|  |  |  | |  | |  | |  |
| *Fixed effects* |  |  | |  | |  | |  |
|  | *b* | *SE* | | *F* | | *df* | | *p* |
| **(Intercept)** | **9.51** | **1.14** | | **69.22** | | **1, 26.55** | | **<.001** |
| **Feedback Timing** | **-5** | **1.97** | | **6.48** | | **1, 38.95** | | **.015** |
| Response Type | 1.09 | 0.79 | | 1.94 | | 1, 24.55 | | .176 |
| Outcome Valence | 0.48 | 0.78 | | 0.37 | | 1, 34.96 | | .546 |
| \|Q\| | 2.82 | 1.49 | | 3.57 | | 1, 28.27 | | .069 |
| Feedback Timing:Response Type | -0.84 | 1.57 | | 0.29 | | 1, 24.55 | | .597 |
| Feedback Timing:Outcome Valence | -2.79 | 1.56 | | 3.20 | | 1, 34.96 | | .082 |
| Response Type:Outcome Valence | -0.83 | 1.13 | | 0.54 | | 1, 23.65 | | .470 |
| Feedback Timing:\|Q\| | -7.28 | 2.98 | | 5.97 | | 1, 28.27 | | .021 |
| Response Type:\|Q\| | 1.17 | 2.05 | | 0.32 | | 1, 29.51 | | .573 |
| **Outcome Valence:\|Q\|** | **7.46** | **1.6** | | **21.79** | | **1, 31.26** | | **<.001** |
| Feedback Timing:Response Type:Outcome Valence | -3.25 | 2.25 | | 2.08 | | 1, 23.65 | | .162 |
| Feedback Timing:Response Type:\|Q\| | -7.69 | 4.11 | | 3.51 | | 1, 29.51 | | .071 |
| **Feedback Timing:Outcome Valence:\|Q\|** | **-11.22** | **3.2** | | **12.34** | | **1, 31.26** | | **.001** |
| Response Type:Outcome Valence:\|Q\| | 1.7 | 3.27 | | 0.27 | | 1, 25.65 | | .608 |
| Feedback Timing:Response Type:Outcome Valence:\|Q\| | 0.89 | 6.53 | | 0.02 | | 1, 25.65 | | .893 |
|  |  | | | | | | | |
| *Random effects* |  | | | | | | | |
|  | Variance | SD | | Correlation | | | | |
| Participant (Intercept) | 49.36 | 7.03 | |  | | | | |
| Participant (Outcome Valence) | 22.55 | 4.75 | | 0.13 | | | | |
| Participant (\|Q\|) | 92.56 | 9.62 | | 0.48 0.60 | | | | |
| Participant (Response Type) | 22.22 | 4.71 | | 0.51 0.25 0.78 | | | | |
| Participant (Outcome Valence:\|Q\|) | 68.70 | 8.29 | | 0.27 0.64 0.49 0.36 | | | | |
| Participant (\|Q\|:Response Type) | 149.14 | 12.21 | | 0.28 0.43 0.71 0.63 0.41 | | | | |
| Participant (Outcome Valence:Response Type) | 32.78 | 5.73 | | 0.29 0.44 0.70 0.63 0.59 0.77 | | | | |
| Participant (Outcome Valence:\|Q\|:Response Type) | 324.13 | 18.00 | | 0.07 -0.01 0.45 0.68 0.00 0.49 0.67 | | | | |
| Electrode (Intercept) | 1.36 | 1.17 | |  | | | | |
|  |  | | | | | | | |
| *Goodness of fit* |  | | | | | | | |
|  | AIC | | BIC | | | | LL | |
|  | 126282.9 | | 126699.2 | | | | -63087.45 | |
|  | Marginal R^2^ | | | | Conditional R^2^ | | | |
|  | 0.033 | | | | 0.346 | | | |
| *Note:* Abbreviations: SE = standard error, SD = standard deviation; AIC = Akaike information criterion; BIC = Bayesian information criterion; LL = log-likelihood; \|Q\| = absolute state-action value derived from the winning reinforcement learning model. Significant effects are highlighted in bold. | | | | | | | | |

# Table S7

| *Model-Based Linear Mixed-Effects Model (LMM) Results on P3 Data* | | | | | | | | | | | | | | | |  |
| --- | --- | --- | --- | --- | --- | --- | --- | --- | --- | --- | --- | --- | --- | --- | --- | --- |
|  | *fronto-central P3* | | | | | | |  | *centro-parietal P3* | | | | | | | |
| *Model formula* |  | | | | | | |  |  | | | | | | | |
|  | P3a ~ 1 + Feedback Timing + Response Type + Feedback Timing:Response Type + Outcome Valence + Feedback Timing:Outcome Valence + Response Type:Outcome Valence + Feedback Timing:Response Type:Outcome Valence + \|Q\| + Feedback Timing:\|Q\| + Response Type:\|Q\| + Feedback Timing:Response Type:\|Q\| + Outcome Valence:\|Q\| + Feedback Timing:Outcome Valence:\|Q\| + Response Type:Outcome Valence:\|Q\| + Feedback Timing:Response Type:Outcome Valence:\|Q\| + (1 + Outcome Valence + \|Q\| + Response Type + Outcome Valence:\|Q\| + Response Type:\|Q\| + Response Type:Outcome Valence + Response Type:Outcome Valence:\|Q\| \| Participant) + (1 \| Electrode) | | | | | | |  | P3b ~ 1 + Feedback Timing + Response Type + Feedback Timing:Response Type + Outcome Valence + Feedback Timing:Outcome Valence + Response Type:Outcome Valence + Feedback Timing:Response Type:Outcome Valence + \|Q\| + Feedback Timing:\|Q\| + Response Type:\|Q\| + Feedback Timing:Response Type:\|Q\| + Outcome Valence:\|Q\| + Feedback Timing:Outcome Valence:\|Q\| + Response Type:Outcome Valence:\|Q\| + Feedback Timing:Response Type:Outcome Valence:\|Q\| + (1 + Outcome Valence + \|Q\| + Response Type + Outcome Valence:\|Q\| + Response Type:\|Q\| + Response Type:Outcome Valence \| Participant) + (1 \| Electrode) | | | | | | | |
|  |  | | | | | | |  |  | | | | | | | |
| *Sampling units* |  | | | | | | |  |  | | | | | | | |
|  | N observations = 16452 | | | | | | |  | N observations =16452 | | | | | | | |
|  | N participants = 56 (28 immediate, 28 delayed feedback) | | | | | | |  | N participants = 56 (28 immediate, 28 delayed feedback) | | | | | | | |
|  | N electrodes = 4 (Fz, FCz, FC1, FC2) | | | | | | |  | N electrodes = 4 (Cz, CP1, CP2, Pz) | | | | | | | |
|  |  | | | | | | |  |  | | | | | | | |
| *Fixed effects* |  | | | | | | |  |  | | | | | | | |
|  | *b* | *SE* | | *F* | *df* | | *p* |  | *b* | *SE* | | *F* | | *df* | *p* | |
| **(Intercept)** | **14.04** | **1.31** | | **115.54** | **1, 14.75** | | **<.001** |  | **17.21** | **1.06** | | **261.47** | | **1, 46.11** | **<.001** | |
| **Feedback Timing** | **-9.13** | **1.99** | | **21.07** | **1, 46.27** | | **<.001** |  | **-6.14** | **2.08** | | **8.69** | | **1, 44.07** | **.005** | |
| Response Type | 1.14 | 0.77 | | 2.21 | 1, 20.98 | | .152 |  | 0.68 | 0.87 | | 0.61 | | 1, 24.02 | .443 | |
| **Outcome Valence** | **4.5** | **0.7** | | **40.94** | **1, 29.98** | | **<.001** |  | **4.55** | **0.86** | | **27.87** | | **1, 30.84** | **<.001** | |
| **\|Q\|** | **-5.12** | **1.32** | | **14.97** | **1, 17.42** | | **.001** |  | **-3.64** | **1.59** | | **5.22** | | **1, 29.71** | **.030** | |
| Feedback Timing:Response Type | -1.41 | 1.54 | | 0.84 | 1, 20.98 | | .370 |  | -1.39 | 1.74 | | 0.64 | | 1, 24.02 | .431 | |
| **Feedback Timing:Outcome Valence** | **-5.09** | **1.41** | | **13.08** | **1, 29.98** | | **.001** |  | **-4.05** | **1.72** | | **5.51** | | **1, 30.84** | **.025** | |
| Response Type:Outcome Valence | 0.25 | 1.34 | | 0.04 | 1, 12.44 | | .853 |  | 0.84 | 1.2 | | 0.49 | | 1, 40.79 | .489 | |
| Feedback Timing:\|Q\| | -1.93 | 2.65 | | 0.53 | 1, 17.42 | | .475 |  | -2.62 | 3.18 | | 0.68 | | 1, 29.71 | .417 | |
| Response Type:\|Q\| | 0.72 | 1.89 | | 0.15 | 1, 19.06 | | .707 |  | 1.54 | 2.63 | | 0.34 | | 1, 27.08 | .562 | |
| **Outcome Valence:\|Q\|** | **11.42** | **2.38** | | **22.98** | **1, 26.63** | | **<.001** |  | **12.13** | **2.54** | | **22.89** | | **1, 43.5** | **<.001** | |
| Feedback Timing:Response Type:Outcome Valence | -1.92 | 2.69 | | 0.51 | 1, 12.44 | | .488 |  | -4.59 | 2.41 | | 3.64 | | 1, 40.79 | .063 | |
| Feedback Timing:Response Type:\|Q\| | -3.77 | 3.77 | | 1.00 | 1, 19.06 | | .331 |  | -9.1 | 5.25 | | 3.00 | | 1, 27.08 | .095 | |
| **Feedback Timing:Outcome Valence:\|Q\|** | **-12.78** | **4.76** | | **7.19** | **1, 26.63** | | **.012** |  | -6.05 | 5.07 | | 1.42 | | 1, 43.5 | .239 | |
| **Response Type:Outcome Valence:\|Q\|** | 6.21 | 4.84 | | 1.65 | 1, 16.91 | | .217 |  | **6.68** | **2.17** | | **9.48** | | **1, 1237.96** | **.002** | |
| **Feedback Timing:Response Type:Outcome Valence:\|Q\|** | -10.14 | 9.68 | | 1.10 | 1, 16.91 | | .310 |  | **-9.58** | **4.34** | | **4.88** | | **1, 1237.96** | **.027** | |
|  |  |  | |  |  | |  |  |  |  | |  | |  |  | |
| *Random effects* |  |  | |  |  | |  |  |  |  | |  | |  |  | |
|  | Variance | SD | | Correlation | | | |  | Variance | SD | | Correlation | | | | |
| Participant (Intercept) | 50.46 | 7.10 | |  | | | |  | 55.12 | 7.42 | |  | | | | |
| Participant (Outcome Valence) | 16.47 | 4.06 | | 0.23 | | | |  | 26.83 | 5.18 | | 0.30 | | | | |
| Participant (\|Q\|) | 60.39 | 7.77 | | 0.25 0.12 | | | |  | 100.18 | 10.01 | | 0.38 0.32 | | | | |
| Participant (Response Type) | 19.37 | 4.40 | | -0.02 0.60 0.46 | | | |  | 25.95 | 5.09 | | 0.19 0.63 0.58 | | | | |
| Participant (Outcome Valence:\|Q\|) | 186.64 | 13.66 | | 0.25 0.41 0.02 0.34 | | | |  | 252.45 | 15.89 | | 0.25 0.47 -0.11 0.10 | | | | |
| Participant (\|Q\|:Response Type) | 76.08 | 8.72 | | -0.13 0.13 0.21 0.45 -0.02 | | | |  | 245.27 | 15.66 | | 0.24 0.46 0.70 0.73 -0.04 | | | | |
| Participant (Outcome Valence:Response Type) | 51.02 | 7.14 | | -0.14 -0.06 -0.31 0.42 -0.07 0.18 | | | |  | 40.96 | 6.40 | | -0.01 0.12 0.01 0.32 -0.29 0.41 | | | | |
| Participant (Outcome Valence:\|Q\|:Response Type) | 767.79 | 27.71 | | 0.14 0.13 0.14 0.55 0.40 -0.10 0.46 | | | |  | - | - | | - | | | | |
| Electrode (Intercept) | 2.87 | 1.69 | |  | | | |  | 0.19 | 0.44 | |  | | | | |
|  |  | | | | | | |  |  | | | | | | | |
| *Goodness of fit* |  | | | | | | |  |  | | | | | | | |
|  | AIC | | BIC | | | LL | |  | AIC | | BIC | | LL | | | |
|  | 131972.1 | | 132388.3 | | | -65932.03 | |  | 131578.2 | | 131932.8 | | -65743.12 | | | |
|  | Marginal R^2^ | | | Conditional R^2^ | | | |  | Marginal R^2^ | | | Conditional R^2^ | | | | |
|  | 0.099 | | | 0.359 | | | |  | 0.063 | | | 0.352 | | | | |
| *Note:* Abbreviations: SE = standard error, SD = standard deviation; AIC = Akaike information criterion; BIC = Bayesian information criterion; LL = log-likelihood; \|Q\| = absolute state-action value derived from the winning reinforcement learning model. Significant effects are highlighted in bold. | | | | | | | | | | | | | | | |  |

# Table S8

| *Model-Free Linear Mixed-Effects Model (LMM) Results on FRN/RewP Data* | | | | | | | | |
| --- | --- | --- | --- | --- | --- | --- | --- | --- |
| *Model formula* |  |  | |  | |  | |  |
|  | FRN/RewP amplitude ~ 1 + Feedback Delay + Response Type + Feedback Delay:Response Type + Outcome Valence + Feedback Delay:Outcome Valence + Response Type:Outcome Valence + Feedback Delay:Response Type:Outcome Valence + (1 + Outcome Valence + Response Type + Response Type:Outcome Valence \| Participant) + (1 \| Electrode) | | | | | | | |
|  | | | | | | | | |
| *Sampling units* |  | | | | | | | |
|  | N observations = 16452 | | | | | | | |
|  | N participants = 56 (28 immediate, 28 delayed feedback) | | | | | | | |
|  | N electrodes = 4 (Fz, FCz, FC1, FC2) | | | | | | | |
|  | | | | | | | | |
| *Fixed effects* |  | | | | | | | |
|  | *b* | *SE* | | *F* | | *df* | | *p* |
| **Intercept** | **8.30** | **1.00** | | **69.16** | | **1, 20.40** | | **<.001** |
| Feedback Delay | -2.88 | 1.62 | | 3.17 | | 1, 52.23 | | .081 |
| Response Type | 0.35 | 0.40 | | 0.79 | | 1, 41.90 | | .379 |
| **Outcome Valence** | **-1.35** | **0.52** | | **6.70** | | **1, 46.17** | | **.013** |
| Feedback Delay:Response Type | 0.52 | 0.8 | | 0.43 | | 1, 41.90 | | .514 |
| Feedback Delay:Outcome Valence | 0.64 | 1.04 | | 0.38 | | 1, 46.17 | | .541 |
| Response Type:Outcome Valence | -1.08 | 0.68 | | 2.52 | | 1, 38.92 | | .120 |
| Feedback Delay:Response Type:Outcome Valence | -1.56 | 1.36 | | 1.30 | | 1, 38.92 | | .260 |
|  |  | | | | | | | |
| *Random effects* |  | | | | | | | |
|  | Variance | SD | | Correlation | | | | |
| Participant (Intercept) | 35.65 | 5.97 | |  | | | | |
| Participant (Outcome Valence) | 10.97 | 3.31 | | -0.18 | | | | |
| Participant (Response Type) | 4.75 | 2.18 | | 0.32 -0.21 | | | | |
| Participant (Outcome Valence:Response Type) | 9.76 | 3.12 | | -0.02 0.17 -0.08 | | | | |
| Electrode (Intercept) | 1.36 | 1.17 | |  | | | | |
|  |  | | | | | | | |
| *Goodness of fit* |  | | | | | | | |
|  | AIC | | BIC | | | | LL | |
|  | 126676.6 | | 126830.8 | | | | -63318.3 | |
|  | Marginal R^2^ | | | | Conditional R^2^ | | | |
|  | 0.018 | | | | 0.255 | | | |
| *Note:* Abbreviations: SE = standard error, SD = standard deviation; AIC = Akaike information criterion; BIC = Bayesian information criterion; LL = log-likelihood. Significant effects are highlighted in bold. | | | | | | | | |

# Table S9

| *Model-Free Linear Mixed-Effects Model (LMM) Results on P3 Data* | | | | | | | | | | | | | | | |
| --- | --- | --- | --- | --- | --- | --- | --- | --- | --- | --- | --- | --- | --- | --- | --- |
|  | *fronto-central P3* | | | | | | |  | *centro-parietal P3* | | | | | | |
| *Model formula* |  | | | | | | |  |  | | | | | | |
|  | P3a ~ 1 + Feedback Timing + Response Type + Feedback Timing:Response Type + Outcome Valence + Feedback Timing:Outcome Valence + Response Type:Outcome Valence + Feedback Timing:Response Type:Outcome Valence + (1 + Outcome Valence + Response Type + Response Type:Outcome Valence \| Participant) + (1 \| Electrode) | | | | | | |  | P3b ~ 1 + Feedback Timing + Response Type + Feedback Timing:Response Type + Outcome Valence + Feedback Timing:Outcome Valence + Response Type:Outcome Valence + Feedback Timing:Response Type:Outcome Valence + (1 + Outcome Valence + Response Type + Response Type:Outcome Valence \| Participant) + (1 \| Electrode) | | | | | | |
|  |  | | | | | | |  |  | | | | | | |
| *Sampling units* |  | | | | | | |  |  | | | | | | |
|  | N observations = 16452 | | | | | | |  | N observations = 16452 | | | | | | |
|  | N participants = 56 (28 immediate, 28 delayed feedback) | | | | | | |  | N participants = 56 (28 immediate, 28 delayed feedback) | | | | | | |
|  | N electrodes = 4 (Fz, FCz, FC1, FC2) | | | | | | |  | N electrodes = 4 (Cz, CP1, CP2, Pz) | | | | | | |
|  |  | | | | | | |  |  | | | | | | |
| *Fixed effects* |  | | | | | | |  |  | | | | | | |
|  | *b* | *SE* | | *F* | *df* | | *p* |  | *b* | *SE* | | *F* | | *df* | *p* |
| **(Intercept)** | **14.53** | **1.23** | | **138.86** | **1, 12.29** | | **<.001** |  | **17.45** | **0.92** | | **360.73** | | **1, 52.79** | **<.001** |
| **Feedback Timing** | **-7.07** | **1.79** | | **15.50** | **1, 53.03** | | **<.001** |  | **-4.56** | **1.79** | | **6.52** | | **1, 51.28** | **.014** |
| Response Type | 0.75 | 0.57 | | 1.70 | 1, 42.73 | | .199 |  | 0.27 | 0.59 | | 0.20 | | 1, 42.22 | .654 |
| **Outcome Valence** | **2.80** | **0.75** | | **13.92** | **1, 43.63** | | **.001** |  | **2.38** | **0.76** | | **9.83** | | **1, 46.94** | **.003** |
| Feedback Timing:Response Type | 1.26 | 1.14 | | 1.22 | 1, 42.73 | | .276 |  | 1.47 | 1.19 | | 1.53 | | 1, 42.22 | .222 |
| **Feedback Timing:Outcome Valence** | **-4.19** | **1.50** | | **7.78** | **1, 43.63** | | **.008** |  | **-3.46** | **1.52** | | **5.18** | | **1, 46.94** | **.027** |
| Response Type:Outcome Valence | -1.12 | 0.99 | | 1.28 | 1, 34.09 | | .266 |  | -1.23 | 1.12 | | 1.20 | | 1, 36.63 | .281 |
| Feedback Timing:Response Type:Outcome Valence | -0.69 | 1.98 | | 0.12 | 1, 34.09 | | .730 |  | -1.73 | 2.25 | | 0.59 | | 1, 36.63 | .446 |
|  |  | | | | | | |  |  | | | | | | |
| *Random effects* |  | | | | | | |  |  | | | | | | |
|  | Variance | SD | | Correlation | | | |  | Variance | SD | | Correlation | | | |
| Participant (Intercept) | 43.16 | 6.57 | |  | | | |  | 42.47 | 6.52 | |  | | | |
| Participant (Outcome Valence) | 23.82 | 4.88 | | 0.08 | | | |  | 23.67 | 4.87 | | -0.01 | | | |
| Participant (Response Type) | 10.77 | 3.28 | | -0.15 -0.11 | | | |  | 11.37 | 3.37 | | -0.04 0.01 | | | |
| Participant (Outcome Valence:Response Type) | 25.08 | 5.01 | | -0.09 0.29 -0.13 | | | |  | 37.17 | 6.10 | | -0.11 0.29 -0.17 | | | |
| Electrode (Intercept) | 2.86 | 1.69 | |  | | | |  | 0.19 | 0.44 | |  | | | |
|  |  | | | | | | |  |  | | | | | | |
| *Goodness of fit* |  | | | | | | |  |  | | | | | | |
|  | AIC | | BIC | | | LL | |  | AIC | | BIC | | LL | | |
|  | 132522.6 | | 132676.8 | | | -66241.31 | |  | 132072.9 | | 132227.1 | | -66016.46 | | |
|  | Marginal R^2^ | | | Conditional R^2^ | | | |  | Marginal R^2^ | | | Conditional R^2^ | | | |
|  | 0.061 | | | 0.282 | | | |  | 0.032 | | | 0.254 | | | |
| *Note:* Abbreviations: SE = standard error, SD = standard deviation; AIC = Akaike information criterion; BIC = Bayesian information criterion; LL = log-likelihood. Significant effects are highlighted in bold. | | | | | | | | | | | | | | | |

# Table S10

| *Generalized Linear Mixed-Effects Model (GLMM) Results on Brain-Behavior Relationships* | | | | | | |
| --- | --- | --- | --- | --- | --- | --- |
| *Model formula* |  | | | | | |
|  | *next-trial p(go) ~ 1 + Feedback Timing + Outcome Valence + Feedback Timing:Outcome Valence + FRN/RewP + Feedback Timing:FRN/RewP + Outcome Valence:FRN/RewP + Feedback Timing:Outcome Valence:FRN/RewP + (1 + Outcome Valence + FRN/RewP + Outcome Valence:FRN/RewP \| Participant)* | | | | | |
|  |  | | | | | |
| *Sampling units* |  | | | | | |
|  | N observations = 17208 | | | | | |
|  | N participants = 56 (28 immediate, 28 delayed feedback) | | | | | |
|  |  | | | | | |
| *Fixed effects* |  | | | | | |
|  | *b* | *SE* | *χ2* | | *df* | *p* |
| **(Intercept)** | **0.75** | **0.09** | **77.65** | | **1** | **<.001** |
| Feedback Timing | -0.15 | 0.17 | 0.8 | | 1 | .370 |
| **Outcome Valence** | **-1.16** | **0.2** | **34.36** | | **1** | **<.001** |
| FRN/RewP | -0.04 | 0.03 | 2.89 | | 1 | .089 |
| Feedback Timing:Outcome Valence | 0.29 | 0.39 | 0.56 | | 1 | .456 |
| Feedback Timing:FRN/RewP | -0.09 | 0.05 | 3.72 | | 1 | .054 |
| Outcome Valence:FRN/RewP | 0.04 | 0.08 | 0.23 | | 1 | .628 |
| Feedback Timing:Outcome Valence:FRN/RewP | -0.13 | 0.16 | 0.74 | | 1 | .391 |
|  |  | | | | | |
| *Random effects* |  | | | | | |
|  | Variance | SD | Correlation | | | |
| Participant (Intercept) | 0.38 | 0.61 |  | | | |
| Participant (Outcome Valence) | 2.06 | 1.44 | -0.86 | | | |
| Participant (FRN/RewP) | 0.02 | 0.13 | -0.05 0.05 | | | |
| Participant (Outcome Valence:FRN/RewP) | 0.27 | 0.52 | 0.33 -0.28 0.21 | | | |
|  |  | | | | | |
| *Goodness of fit* |  | | | | | |
|  | AIC | BIC | LL | | Deviance | |
|  | 20536.2 | 20675.7 | -10250.1 | | 20500.2 | |
|  | Marginal R^2^ | | | Conditional R^2^ | | |
|  | 0.073 | | | 0.306 | | |
| *Note:* Abbreviations: SE = standard error, SD = standard deviation; AIC = Akaike information criterion; BIC = Bayesian information criterion; LL = log-likelihood; Significant effects are highlighted in bold. | | | | | | |

# Table S11

| *Generalized Linear Mixed-Effects Model (GLMM) Results on Brain-Behavior Relationships* | | | | | | | | | | | | | | | | | |
| --- | --- | --- | --- | --- | --- | --- | --- | --- | --- | --- | --- | --- | --- | --- | --- | --- | --- |
|  | *fronto-central P3 (P3a)* | | | | | | | | |  | *centro-parietal P3 (P3b)* | | | | | | |
| *Model formula* |  | | | | | | | | |  |  | | | | | | |
|  | *next trial p(go) ~ 1 + Feedback Timing + Outcome Valence + Feedback Timing:Outcome Valence + P3 + Feedback Timing:P3 + Outcome Valence:P3 + Feedback Timing:Outcome Valence:P3 + (1 + Outcome Valence + P3 + Outcome Valence:P3 \| Participant)* | | | | | | | | |  | *next trial p(go) ~ 1 + Feedback Timing + Outcome Valence + Feedback Timing:Outcome Valence + P3 + Feedback Timing:P3 + Outcome Valence:P3 + Feedback Timing:Outcome Valence:P3 + (1 + Outcome Valence + P3 + Outcome Valence:P3 \| Participant)* | | | | | | |
|  |  | | | | | | | | |  |  | | | | | | |
| *Sampling units* |  | | | | | | | | |  |  | | | | | | |
|  | N observations = 17208 | | | | | | | | |  | N observations = 17208 | | | | | | |
|  | N participants = 56 (28 immediate, 28 delayed feedback) | | | | | | | | |  | N participants = 56 (28 immediate, 28 delayed feedback) | | | | | | |
|  |  | | | | | | | | |  |  | | | | | | |
| *Fixed effects* |  | | | | | | | | |  |  | | | | | | |
|  | *b* | *SE* | | *χ2* | | | *df* | | *p* |  | *b* | *SE* | *χ2* | | *df* | | *p* |
| **(Intercept)** | **0.75** | **0.08** | | **79.11** | | | **1** | | **<.001** |  | **0.75** | **0.08** | **77.89** | | **1** | | **<.001** |
| Feedback Timing | -0.13 | 0.17 | | 0.64 | | | 1 | | .423 |  | -0.13 | 0.17 | 0.56 | | 1 | | .452 |
| **Outcome Valence** | **-1.15** | **0.2** | | **33.33** | | | **1** | | **<.001** |  | **-1.15** | **0.2** | **33.63** | | **1** | | **<.001** |
| **P3** | **-0.09** | **0.03** | | **8.88** | | | **1** | | **.003** |  | **-0.06** | **0.03** | **4.34** | | **1** | | **.037** |
| Feedback Timing:Outcome Valence | 0.23 | 0.4 | | 0.34 | | | 1 | | .560 |  | 0.26 | 0.39 | 0.45 | | 1 | | .502 |
| Feedback Timing:P3 | -0.07 | 0.06 | | 1.38 | | | 1 | | .240 |  | -0.08 | 0.06 | 2.29 | | 1 | | .130 |
| Outcome Valence:P3 | 0.04 | 0.05 | | 0.66 | | | 1 | | .417 |  | 0 | 0.06 | 0 | | 1 | | .983 |
| Feedback Timing:Outcome Valence:P3 | 0.12 | 0.1 | | 1.6 | | | 1 | | .206 |  | 0.1 | 0.11 | 0.83 | | 1 | | .363 |
|  |  |  | |  | | |  | |  |  |  |  |  | |  | |  |
| *Random effects* |  |  | |  | | |  | |  |  |  |  |  | |  | |  |
|  | Variance | SD | | Correlation | | | | | |  | Variance | SD | Correlation | | | | |
| Participant (Intercept) | 0.37 | 0.61 | |  | | | | | |  | 0.37 | 0.61 |  | | | | |
| Participant (Outcome Valence) | 2.08 | 1.44 | | -0.86 | | | | | |  | 2.08 | 1.44 | -0.85 | | | | |
| Participant (P3) | 0.03 | 0.19 | | -0.08 0.05 | | | | | |  | 0.03 | 0.17 | -0.17 0.07 | | | | |
| Participant (Outcome Valence:P3) | 0.09 | 0.29 | | 0.14 -0.23 0.06 | | | | | |  | 0.11 | 0.34 | 0.08 -0.13 -0.13 | | | | |
|  |  | | | | | | | | |  |  | | | | | | |
| *Goodness of fit* |  | | | | | | | | |  |  | | | | | | |
|  | AIC | | BIC | | LL | | | Deviance | |  | AIC | BIC | | LL | | Deviance | |
|  | 20478.8 | | 20618.3 | | -10221.4 | | | 20442.8 | |  | 20490.5 | 20630.1 | | -10227.3 | | 20454.5 | |
|  | Marginal R^2^ | | | | | Conditional R^2^ | | | |  | Marginal R^2^ | | | Conditional R^2^ | | | |
|  | 0.076 | | | | | 0.310 | | | |  | 0.073 | | | 0.309 | | | |
| *Note:* Abbreviations: SE = standard error, SD = standard deviation; AIC = Akaike information criterion; BIC = Bayesian information criterion; LL = log-likelihood; Significant effects are highlighted in bold. | | | | | | | | | | | | | | | | | |
